# pLMSNOSite: an ensemble-based approach for predicting protein S-nitrosylation sites by integrating supervised word embedding and embedding from pre-trained protein language model

**DOI:** 10.1186/s12859-023-05164-9

**Published:** 2023-02-08

**Authors:** Pawel Pratyush, Suresh Pokharel, Hiroto Saigo, Dukka B. KC

**Affiliations:** 1 Department of Computer Science, Michigan Technological University, Houghton, MI USA; 2 Department of Electrical Engineering and Computer Science, Kyushu University, 744, Motooka, Nishi-Ku, 819-0395 Japan

**Keywords:** S-nitrosylation, Deep learning, Convolutional neural network, Post-translational modification, Word embedding, Protein language model

## Abstract

**Background:**

Protein S-nitrosylation (SNO) plays a key role in transferring nitric oxide-mediated signals in both animals and plants and has emerged as an important mechanism for regulating protein functions and cell signaling of all main classes of protein. It is involved in several biological processes including immune response, protein stability, transcription regulation, post translational regulation, DNA damage repair, redox regulation, and is an emerging paradigm of redox signaling for protection against oxidative stress. The development of robust computational tools to predict protein SNO sites would contribute to further interpretation of the pathological and physiological mechanisms of SNO.

**Results:**

Using an intermediate fusion-based stacked generalization approach, we integrated embeddings from supervised embedding layer and contextualized protein language model (ProtT5) and developed a tool called pLMSNOSite (protein language model-based SNO site predictor). On an independent test set of experimentally identified SNO sites, pLMSNOSite achieved values of 0.340, 0.735 and 0.773 for MCC, sensitivity and specificity respectively. These results show that pLMSNOSite performs better than the compared approaches for the prediction of S-nitrosylation sites.

**Conclusion:**

Together, the experimental results suggest that pLMSNOSite achieves significant improvement in the prediction performance of S-nitrosylation sites and represents a robust computational approach for predicting protein S-nitrosylation sites. pLMSNOSite could be a useful resource for further elucidation of SNO and is publicly available at https://github.com/KCLabMTU/pLMSNOSite.

**Supplementary Information:**

The online version contains supplementary material available at 10.1186/s12859-023-05164-9.

## Background

Nitric oxide (NO) is a highly reactive molecule, and abnormal NO levels in mammalian cells are associated with multiple human diseases, including cancer [1]. The role of NO as a major regulator of physiological function has become increasingly evident. S-nitrosylation (SNO) is one of the most important regulatory mechanisms of this vital signaling molecule. In S-nitrosylation, the NO is covalently attached to the thiol side chain of cysteine residues to form S-nitrosothiol (SN), a critical mechanism of transferring NO-mediated signals [2]. Additionally, S-nitrosylation has unfolded as an important mechanism for regulating protein functions and cell signaling of all main classes of protein and is involved in several biological processes including immune response [1], protein stability, transcription regulation, post translational regulation, DNA damage repair, and redox regulation [3], and is an emerging paradigm of redox signaling for protection against oxidative stress. Recently, it has also been shown that SNO also regulates diverse biological processes in plants [4].

The experimental identification of S-nitrosylated sites is generally performed by combining the Biotin-switch technique (BST) [5]with Mass Spectrometry (MS). With few exceptions, all methods for the identification of S-nitrosylation sites are based on the BST and differ only in the utilized MS equipment, ion sources, and the use of liquid chromatography. Please refer to the excellent review by Lamotte et al. [4] for an in-depth description of experimental identification of S-nitrosylation.

Although some studies have suggested that the target cysteine residues often lie within an acid–base or hydrophobic motif [6], recent studies have proven that the acid–base motif is located farther from the cysteine [7]. Additionally, even though some studies have suggested that the target cysteine must be within a signature motif (I/L-X-C-X2-D/E) and be in a suitable environment [1], there is not yet a consensus motif for SNO [8]. In this regard, various mechanisms are involved in the formation of SNO.

Owing to this fact that high throughput experimental approaches do not yet exist for SNO, several complimentary computational approaches have been developed to predict protein SNO sites. These approaches are mostly based on machine learning models that use experimentally identified S-nitrosylation sites to train the model and use various features such as identity of the neighboring residues during training. Some of the existing SNO site prediction tools are: GPS-SNO [9], SNOSite [10], iSNOPSeAAC [11], etc. SNOSID [12], developed by Hao et al., is perhaps the first computational tool for predicting S-nitrosylation sites. GPS-SNO [9] is another approach for prediction of S-nitrosylation sites and is based on the GPS 3.0 algorithm. Moreover, iSNO-PseAAC [11] is another approach developed by Xu et al. that uses PseAAC to represent protein sequences for prediction of protein S-nitrosylation sites. Recently, various deep learning-based methods [13, 14] have been developed for prediction of various post-translation modification sites including SNO sites. In that regard, DeepNitro [15], a deep learning-based approach, developed by Xie et al. for the prediction of protein S-nitrosylation sites uses four different types of features: one-hot encoding, Property Factor Representation (PFR), k-space spectrum, and PSSM encoding.

Additionally, Hasan et al. proposed PreSNO [16] which integrates two classifiers: RF and SVM using Linear regression. The input to both the RF and SVM in PreSNO is based on four different encoding schemes: the composition of profile-based amino acid pair (CPA), the K-space spectral amino acid composition (SAC), tripeptide composition from PSSM (TCP), and physicochemical properties of amino acids (PPA). It must be noted here that the DeepNitro dataset is used for training and testing of the PreSNO model. For a thorough review of the existing computational approaches for predicting Protein S-nitrosylation sites, please refer to Zhao et al. [17].

Lately, we have witnessed the development of exciting array of Natural Language Processing (NLP) algorithms and technologies including recent breakthroughs in the field of bioinformatics [14, 18–20]. Among these developments, language models (LMs) have emerged as a powerful paradigm in NLP for learning embeddings directly from large, unlabeled natural language datasets. In contrast to uncontextualized word embeddings, which return the same embedding for a word irrespective of the surrounding words, embeddings from LMs are contextualized in a way that they render the embedding dependent on the surrounding words. These advances are now being explored in proteins through the development of various protein language models (pLMs) [21–24]. The representations (embeddings) extracted from these transformer-based language models have been successful for various downstream bioinformatics prediction tasks [25–27], suggesting that the huge amount of information learned by these pLMs can be transferred to other tasks by extracting embeddings from these pLMs and using these embeddings as an input to predict other properties of protein.

As discussed above, though there exist various computational approaches for predicting SNO sites, the prediction performance of the existing approaches is not yet satisfactory. Additionally, the potential uses of deep learning methods including natural language processing and language models in predicting SNO sites is largely unexplored. Furthermore, the existing approaches do not leverage the distilled information from these pLMs. To the best of our knowledge, embedding from pLMS has not been previously used to predict SNO sites. In this regard, here we propose pLMSNOSite, a stacked generalization approach based on intermediate fusion of models that combines two different learned marginal amino acid sequence representations: per-residue contextual embedding learned on full sequences from a pre-trained protein language model and per-residue supervised word embedding learned on window sequences. Based on independent testing, pLMSNOSite performs better than other widely available approaches for SNO site prediction in proteins.

## Methods

### Benchmark dataset

The training and testing dataset for this work was adopted from PreSNO [16]. PreSNO utilizes the original DeepNitro [15] dataset which is curated through an extensive literature search for experimentally verified S-nitrosylation sites. This dataset consists of an experimentally confirmed 4762 sites from 3113 protein sequences. These sequences are first subjected to homology removal using the cd-hit algorithm [28] with an identity cut-off of 0.3, resulting in 3734 positive sites. The remaining cysteine residues from the same protein sequences (ones that have the experimental SNO sites) are considered as the negative S-nitrosylation sites resulting in 20,548 negative sites. Furthermore, by eliminating the negative site if there is an identical window sequence in the set of positive sites, we obtained 20,333 negative sites. From these sites, the independent dataset is constructed by randomly sampling 20% of the sites, and the remaining sites are used to construct the training dataset. This resulted in 3383 SNO sites and 17,165 non-SNO sites in the training set and 351 SNO sites and 3168 non-SNO sites in the independent test set. Clearly, the training set is highly skewed in class distribution towards negative sites. This imbalance in the training dataset was resolved by randomly undersampling the negative sites. The balanced training set thus obtained was used for building the models whereas the independent test set was unaltered for assessing the generalization ability of the trained models on unseen data. Note that the main difference between DeepNitro [15] and PreSNO [16] datasets is the different cut-off used in cd-hit [28]. The description of the training dataset and independent dataset used in the study is shown in Tables 1 and 2 respectively.

**Table 1:** Number of proteins, number of sites, and training set used in this study (adopted from PreSNO)

Sites	Number of proteins	Number of sites (before balancing)	Number of sites (after balancing)
SNO sites	1962	3383	3383
Non-SNO sites	340	17,165	3383
Total	2302	20,548	6766

The balanced sites are used for training the model

**Table 2:** Number of proteins, positive, and negative sites of independent test set used in the experiments (adopted from PreSNO)

Sites	Number of proteins	Number of sites
SNO sites	267	351
Non-SNO sites	231	3168
Total	438	3519

### Sequence representation

A critical step before passing amino acid sequences to a machine learning model is the numerical encoding of each amino acid through an encoding scheme that assigns a numerical representation to the amino acid. Choosing informative, discriminating, and independent encoding (or features) is a crucial element of effective machine learning algorithms. Most of the existing SNO prediction tools rely on manual or hand-crafted features for the representation of amino acids [17]. We aim to eliminate the reliance on hand-crafted features by leveraging two feature representation approaches for establishing a robust representation of S-nitrosylation sites: word embeddings from a supervised embedding layer and embeddings from ProtT5 (ProtT5-XL-UniRef50) [21], a pre-trained protein language model based on Google’s T5 (Text-to-Text Transfer Transformer) [29] architecture. Below, we describe these two types of embeddings in detail.

### Word embedding using supervised embedding layer

Word embedding is a class of approaches to represent words using a dense vector representation. Protein sequences can be seen as documents, and amino acids that make the protein sequence can be seen as words. In that regard, amino acids (words) can be represented by dense vectors using word embeddings where a vector represents the projection of the amino acid into a continuous vector space. We used Keras’s embedding layer [30], as in LMSuccSite [27], to implement supervised word embedding where the embedding is learned as a part of training a deep learning model. The process of parameter learning in this approach is supervised; the parameters are updated with subsequent layers during the learning process under the supervision of a label.  With subsequent epochs, the layer learns a feature-rich representation of sequences while still preserving the semantic relation between amino acids (each vectorized representation being orthogonal in some other dimension [31]). The input for this representation is the window sequence centered around the site of interest flanked by an equal number of residues upstream and downstream. In cases where there are not enough residues to create the window sequence, we pad the window with virtual amino acids (‘−’). Initially, the amino acids are integer encoded, so that each amino acid can be represented by a unique integer which is provided as an input to the embedding layer. Then, the embedding layer is initialized with random weights, and the layer will learn better embedding for all the amino acids with subsequent epochs as the part of the training process. There are three salient parameters in word embedding (obtained through Keras’s embedding layer) that determines the quality of the feature representation of amino acid sequences. These parameters are *input_dim* denoting the size of the vocabulary, *output_dim* denoting the length of the feature vector for each word and *input_length* denoting the maximum length of input sequence (in our case, the length of window sequence). The vocabulary size is set to 23 to represent 20 canonical, two non-canonical, and one virtual amino acid (denoted by ‘−’.). Based on fivefold cross-validation on a wide range of values of embedding dimension, we obtained the best performance using a dimension of size four. Similarly, performing fivefold cross-validation on multiple window sizes, we obtained the best results using a window size of 37. Hence, the output of the embedding layer is 37 × 4 where 37 is the window size and four is the embedding dimension. The hyperparameter tuning of the window size (input_length) and the embedding dimension (output_dim) is explained in detail in the result section.

### Embedding from pre-trained protein language model ProtT5

**Fig. 1:**
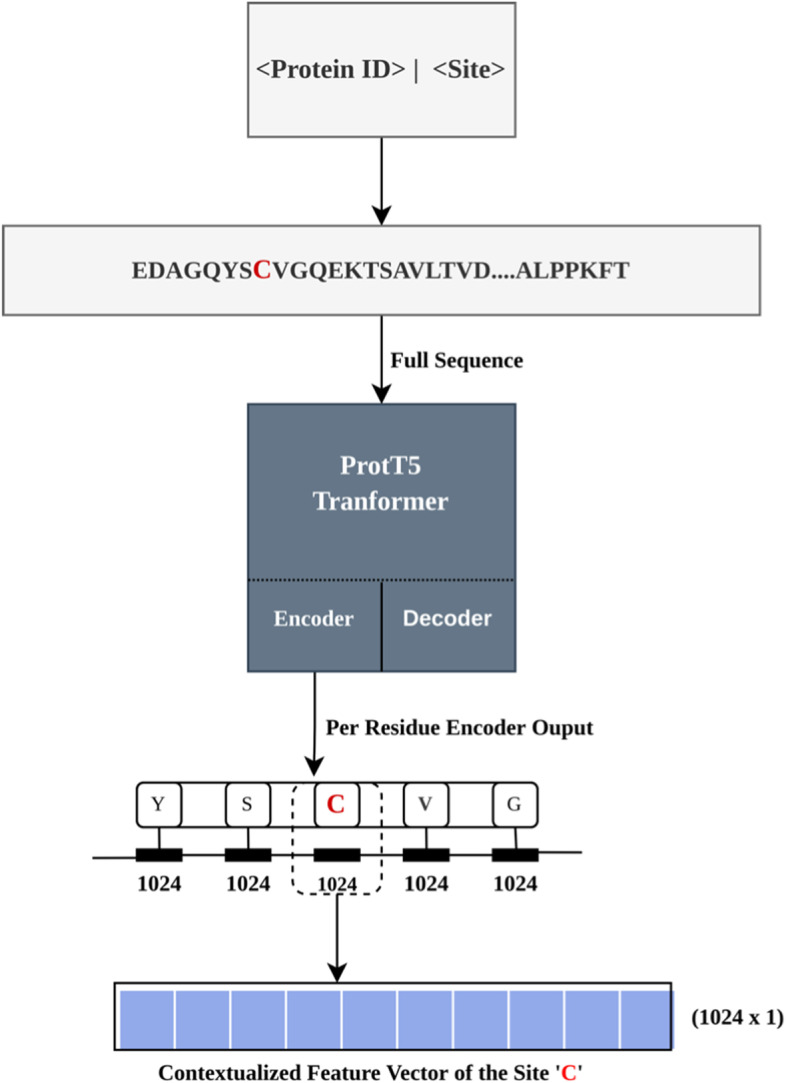
Extraction of Embeddings from ProtT5 language model, the site is the site of interrogation (C, represented in red)

Another representation that we use in our work is based on embeddings from ProtT5, a pre-trained protein language model (pLM). The advances in Natural Language Processing (NLP) gained by the development of newer language models have been transferred to protein sequences by learning to predict masked or missing amino acids using a large corpus of protein sequences [21–23]. Processing/distilling the information learned by these pLMs yields a representation of protein sequences referred to as embeddings [21]. Recently, these embeddings have been shown to be beneficial in various structural bioinformatics tasks including but not limited to secondary structure prediction and subcellular location, among others. In that regard, in this work, we use pLM ProtT5 [21, 27] as a static feature encoders to extract per residue embeddings for protein sequences for which we are predicting S-nitrosylation sites. It is relevant to note that the input to ProtT5 is the overall protein sequence. ProtT5 is a pLM trained on BFD (Big Fantastic Database consisting of 2.5 billion sequences), fine-tuned on Uniref50 consisting of 45 million sequences, and developed at Rostlab using T5 [29] architecture. Infact, postional encoding is learned specific to each attention head in the transfromer architecture which is shared across all the layers of attention stack. Using ProtT5, the per-residue embeddings were extracted from the last hidden layer of the encoder model with the size of *L*x1024, where *L* is the size of the protein using the overall protein sequence as the input. As suggested by ProtTrans [26], LMSuccSite [27], the encoder side of ProtT5 was used, and embeddings were extracted in half-precision. For our purpose, as the per-residue embeddings are a contextualized representation, we only used the 1024 length embeddings for the site of interrogation (aka cystine ‘C’). The schematic of the extraction of embedding from ProtT5 is shown in Fig. 1.

### Deep learning models

Given the input and output, we train several DL models to learn underlying patterns in the protein sequence.

### Sequence-based models

The input to the model is the sequence of amino acids which can thought of as sequence of words in the field of natural language processing (NLP). Hence, an obvious option is to employ models designed to train and process sequences, such as recurrent neural network (RNN), long-short term memory (LSTM) [32], bidirectional long-short term memory (BiLSTM), and so forth. The main drawback of these sequence-oriented models is that they are computationally intense, requiring a large number of parameters for training.

### Convolution neural network (CNN) model

CNN models have demonstrated great success in various computer vision tasks, where convolution kernels or filters are used to learn and discern the spatial co-relation between pixels in images. In our SNO site prediction setting, CNNs can help learn the underlying relationship among the amino acids in the input protein sequence. CNNs are less computationally intensive models than sequence-oriented models and facilitate the training of deeper networks as significantly fewer parameters are needed to be learned. The usage of CNNs is prevalent in several PTM prediction tasks [13, 15, 27]. In our case, we use CNN to process the feature representation of the protein sequence obtained from the word embedding layer as described in the previous section. The process of obtaining feature maps of input integer encoded window sequence from the convolution layer (or kernel) is given by the formula:

1 $$G\left[m,n\right]=\left(f\cdot h\right)\left[m,n\right]={\sum }_{ j}{\sum }_{k }h\left[j,k\right]f\left[m-j,n-k\right]$$

where the input sequence is denoted by *f* and the kernel by *h*. The index of rows and columns in the resultant matrix is denoted by *m* and *n* respectively. Typically, we use multiple convolutions over the input sequence which helps to extract diverse features from a single input map and the output maps are stacked forming a volume. The dimension of the obtained feature map from convolution over volume can be calculated using the following formula:

2 $$\left[n,n,{n}_{c}\right].\left[f,f,{n}_{c}\right]=\left[floor\left(\frac{n+2p-f}{s}+1\right),floor\left(\frac{n+2p-f}{s}+1\right),{n}_{f}\right]$$

where *n* is the size input sequence, *n*_*c*_ is the number of channels, f is the kernel size, *p* is the used padding *s* is the used stride and *n*_*f*_ is the number of kernels. The convolution layer is followed by the max-pooling layer which selects the maximum value from regions of feature maps, creating a downsampled map. The downsampled feature map is then flattened and passed into a conventional fully connected network. All the weights in the network are updated using the backpropagation algorithm. It is to be mentioned that we use a non-linear activation function called ReLU (Rectified Linear Unit) in all layers of the architecture for capturing non-linear signals in the data. Among other activation functions, ReLU is widely adopted in deep learning applications due to its benefits such as representational sparsity and efficiency with respect to computation. The ReLU activation function for a domain value *x* is given by:

3 $$RELU\left(x\right) = \mathrm{max}\left(0,x\right)$$

### pLMSNOSite architecture

**Fig. 2:**
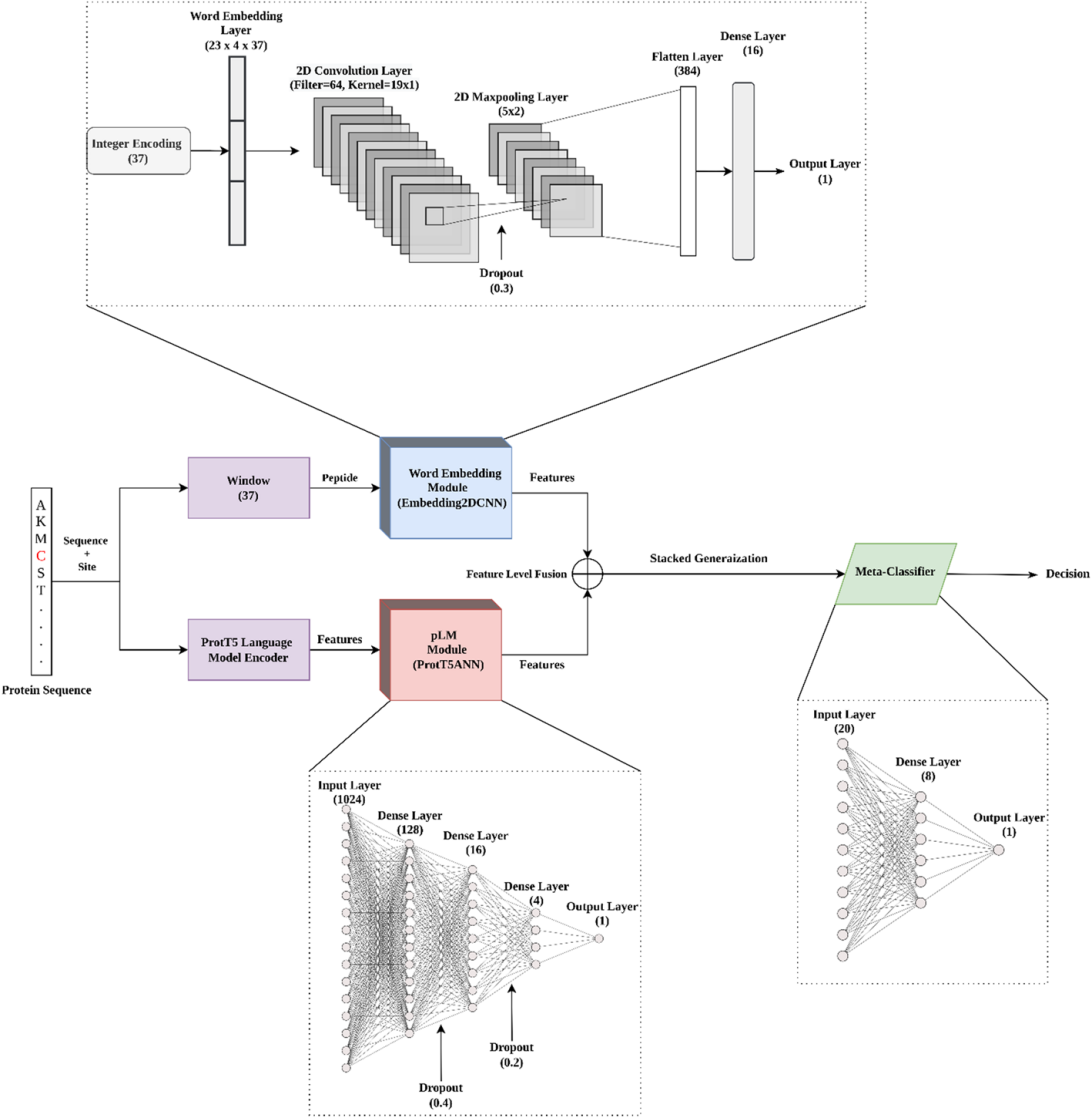
The overall architecture of pLMSNOSite with the two base models and a meta-classifier model

The general framework of the stacked generalization consists of two base models (level-0 models) and a higher-level meta-model (level-1 model, meta-classifier). Our approach pLMSNOSite (protein Language Model-based S-Nitrosylation Site predictor) uses stacked generalization to combine the meta-features (marginal representations) learned from the base models to achieve better prediction. Specifically, the first base model (herein referred to as embedding layer module) learns the representation of local information of cysteine residue of interest captured by proximal residues within window sequences using supervised word embedding. The second base model (herein referred to as pLM ProtT5 module) learns the contextualized information of the same cysteine residue generated by unsupervised pLM using a full-length sequence as input. These learned features by the base models using different representations are fused together and a meta-model is learned adopting an ensemble approach known as stacked generalization. The overall architecture of pLMSNOSite is shown in Fig. 2. As shown in the figure, the architecture of pLMSNOSite consists of two base models: the supervised embedding layer module and the ProtT5 module, followed by a higher-level meta-model (meta-classifier) that performs the feature-level fusion of base models. We further describe the supervised embedding layer module and ProtT5 modules and higher-level meta-model in detail below.

### Supervised (word) embedding layer module

The input to this module is a protein window sequence (centered around the site of interest flanked by an equal number of residues on both sides) that captures the local interaction between amino acids surrounding the site of interrogation (in this case S-nitrosylation/non-S-nitrosylation sites) within the window sequence. We choose a deep two-dimensional (2D) Convolutional Neural Network (CNN) to extract feature maps from these localized interactions of proximal amino acids. The advantage of CNN over other sequence-oriented models has been explained in the previous section. Interestingly, the CNN model also showed promising performance in fivefold cross-validation (refer result section). The 2D-CNN architecture in this module first consists of a word embedding layer which takes integer encoded sequence as an input. The output from this layer (37 × 4, where 37 is the window size and 4 is the embedding dimension) is passed into a 2D convolutional layer to extract feature maps from a window sequence followed by a dropout layer to prevent overfitting, a max-pooling layer and a fully connected layer consisting of a flatten layer and a dense layer. The hyperparameters associated with the model architecture were determined by performing an extensive grid search based on fivefold cross-validation. The search space and optimal hyperparameter values of the model obtained from cross-validation are reported in the Additional file 1: Table S2. Finally, the feature map of size 16 obtained from the final hidden layer from the optimized 2D-CNN model (hereafter dubbed Embedding2DCNN) is treated as the output of the first base model.

### pLM ProtT5 module

In this module, at first per-residue embeddings are extracted from the last hidden layer of the encoder models of ProtT5 of the size of *L*x1024, where *L* is the length of the protein using the overall protein sequence as the input. Subsequently, the 1024 features corresponding to the site of interest are extracted and fed as an input to this module. A dense neural network was used to learn the representation from the obtained features. The architecture of this model and its corresponding hyperparameter values in this module were also chosen based on grid search using fivefold cross-validation. The search space and selected hyperparameter values are reported in the Additional file 1: Table S1. Similar to Embedding2DCNN, we obtained a feature map of size 4 from this base model (hereafter dubbed as ProtT5ANN module).

### Stacked generalization

To integrate the capability of the representation learned by the base models (Embedding2DCNN and ProtT5ANN), we implemented stacked generalization of these modules. To this end, instead of stacking on a decision level or a score level, we performed an intermediate level feature fusion by concatenating the feature maps obtained from the final hidden layers of the base models (16 x 1 from the Embedding2DCNN and 4 x 1 from the ProtT5ANN) as explained in previous subsections. The fused features were then used to train the meta-model (meta-classifier) that acts as the final inference model. Since the datasets used to train base models and meta-classifier are similar, there is a likelihood of data leakage about target information from base models to meta-classifier [33], which could result in overestimation of cross-validation performance leading to spuriousness in the model selection process. Considering this, we paid special attention to ensure that there is no data leakage from base models to meta-classifier. In this work, we performed the fivefold cross-validation algorithm called Stacking with K-fold cross-validation, developed by Wolpert [34], to ensure no target information is leaked while training the meta-classifier. Initially, the overall training data are randomly split into K folds. Subsequently, base models are trained using K-1 folds, and the models are tested against the remaining onefold validation set. The predictions or features obtained from different base models for each fold are collected to train the next-level model (meta classifier). As a result, the meta classifier is trained on a non-overlapping dataset preventing any potential data leakage. Similar to other modules, we selected a single layer feed forward neural network as the architecture for the stacked generalization model using cross-validation.

### Model training

All the deep learning models were trained to minimize the binary cross-entropy loss or log loss function which is given by the following equation:

4 $$-\frac{1}{N}{\sum }_{i=1}^{N}\left[{y}_{i}\mathrm{log}\left({y}_{i}^{^{\prime}}\right)+\left(1-{y}_{i}\right)\mathrm{log}\left(1-{y}_{i}^{^{\prime}}\right)\right]$$

where ${y}_{i}$ and ${y}_{i}^{^{\prime}}$ are the ground truth and predicted probability for the *i*th instance of N points respectively.

The parameters in the model were optimized to minimize the above loss function using Adam stochastic optimization method (AMSGrad variant) with an adaptive learning rate of 0.001, the decay rate for the first moment as 0.9, and the decay rate for the second moment as 0.999. Prior to training, the number of epochs was set to 200 and the batch size was set to 128. Additionally, an early stopping strategy with patience equal to 5 was used which stops the training after 5 epochs if no improvement in loss is recorded. Any potential overfitting while training was averted by carefully monitoring accuracy/loss curves.

### Evaluation of models and performance metrics

We adopt a stratified fivefold cross-validation strategy for model selection. Subsequently, we perform independent testing to assess the generalization error of our approach as well as compare with it the existing approaches. Below, we define the performance metrics used for evaluating the models.

5 $$\mathrm{Accuracy (ACC) }= \frac{TP+TN}{TP+TN+FP+FN}$$

6 $$\mathrm{Sensitivity(SN) }= \frac{TP}{TP+FN}$$

7 $$\mathrm{Specificity(SP) }= \frac{TN}{TN+FP}$$

8 $$\mathrm{MCC }= \frac{TP*TN-FP*FN}{\left(TP+FP\right)\left(TP+FN\right)\left(TN+FP\right)\left(TN+FN\right)}$$

where TP (True Positive) is the number of actual SNO sites predicted as positive, TN (True Negative) is the number of non-SNO sites predicted as negative, FP (False Positive) is the number of non-SNO sites predicted as positive and FN (False Negative) is the number of actual SNO sites predicted as negative.

We also use AUROC (Area Under Receiver Operating Characteristic curve) and AUPR (Area Under Precision-Recall curve) to further evaluate the discriminating performance of the models.

## Results

As described above, pLMSNOSite uses stacked generalization to combine the supervised word embedding layer module (Embedding2DCNN) and the pLM ProtT5 module (ProT5ANN) using a meta-classifier. The meta-classifier in fact learns from the output of the base models and thus the base models were first optimized to robustly learn their corresponding representations. Successively, the meta-classifier was optimized to produce the classification inference accurately.

Initially, we analyze the comparative performance of various ML/DL architectures for the selection of the optimal base models using fivefold cross-validation. Subsequently, the comparative cross-validation performance of various models was analyzed for the selection of optimal meta-classifier. Finally, we compare the performance of the overall architecture pLMNOSite against existing SNO site prediction tools using the independent test set. The details of the results obtained from these experiments are presented in the following subsections.

### Selection of window size and embedding dimension for word embedding module

As described in the Methods section, the supervised embedding layer has three major parameters: vocabulary size (input_dim), window size (input_length), and embedding dimension (output_dim). The *input_dim* is fixed to 23 based on the number of canonical amino acids (= 20), non-canonical amino acids (= 2), and virtual amino acids (= 1). The window size (input_length) is important as too few residues might result in information loss while too many residues might result in loss of local contextual information of the site. To obtain the optimal *input_length*, fivefold cross-validation was performed by varying window sizes from 21 to 63. Similarly, a higher embedding dimension demands substantial computational cost and thus the optimal *output_dim* was determined by exhaustively searching the value of the embedding dimension in the search space ranging from 2 to 32.

**Fig. 3:**
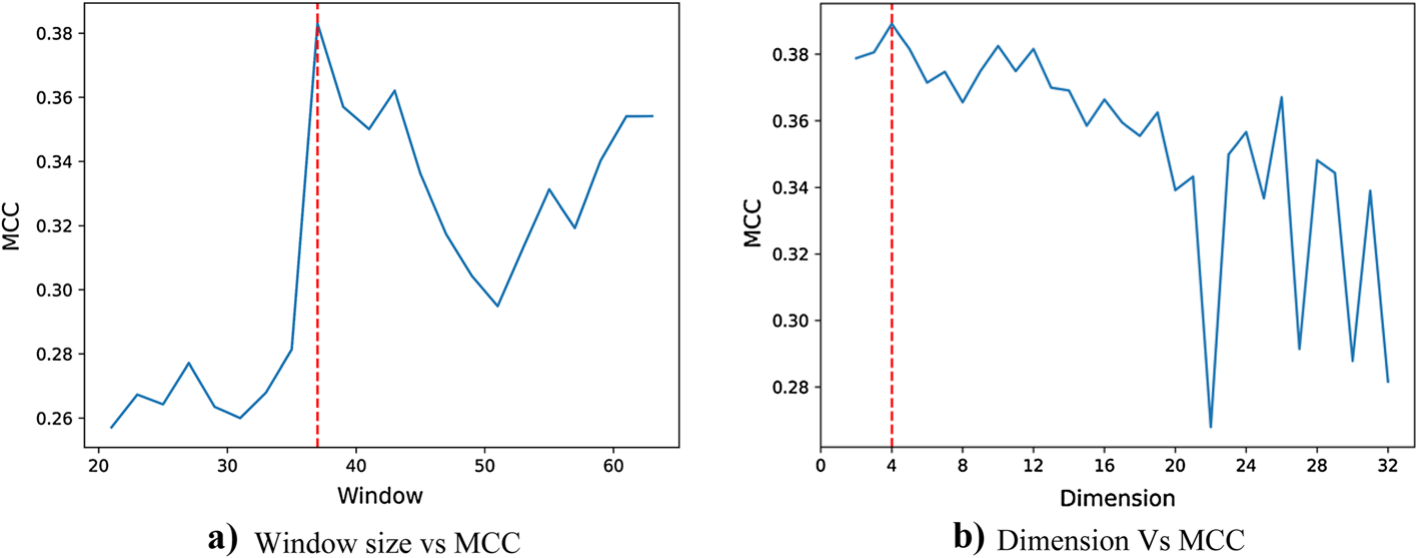
Sensitivity analysis of MCC on fivefold cross-validation when **a** window size is varied keeping the dimension and vocabulary size constant (dimension = 4, vocabulary size = 23), **b** dimension is varied keeping the window size and vocabulary size constant (window size = 37, vocabulary size = 4)

The cross-validation experiments suggest that the output_dim (or, embedding dimension) of 4 and input_length (or window size) of 37 produced the highest MCC and these values were utilized for further analysis. The obtained value of output dimension is indeed a significant improvement over the traditional binarization encoding (or, one-hot encoding) where static and relatively higher dimensional features are generated. It is also worthwhile to note that the optimal window size for PreSNO is 41 (only 2 residue difference on each side of the central residue). The sensitivity analysis of MCC (mean) on fivefold cross-validation for different window sizes and embedding dimension for Embedding2DCNN is shown in Fig. 3a, b respectively and the respective plots for other models are in Additional file 1: Fig. S1.

### Selection of model architecture for the word embedding  module

**Fig. 4:**
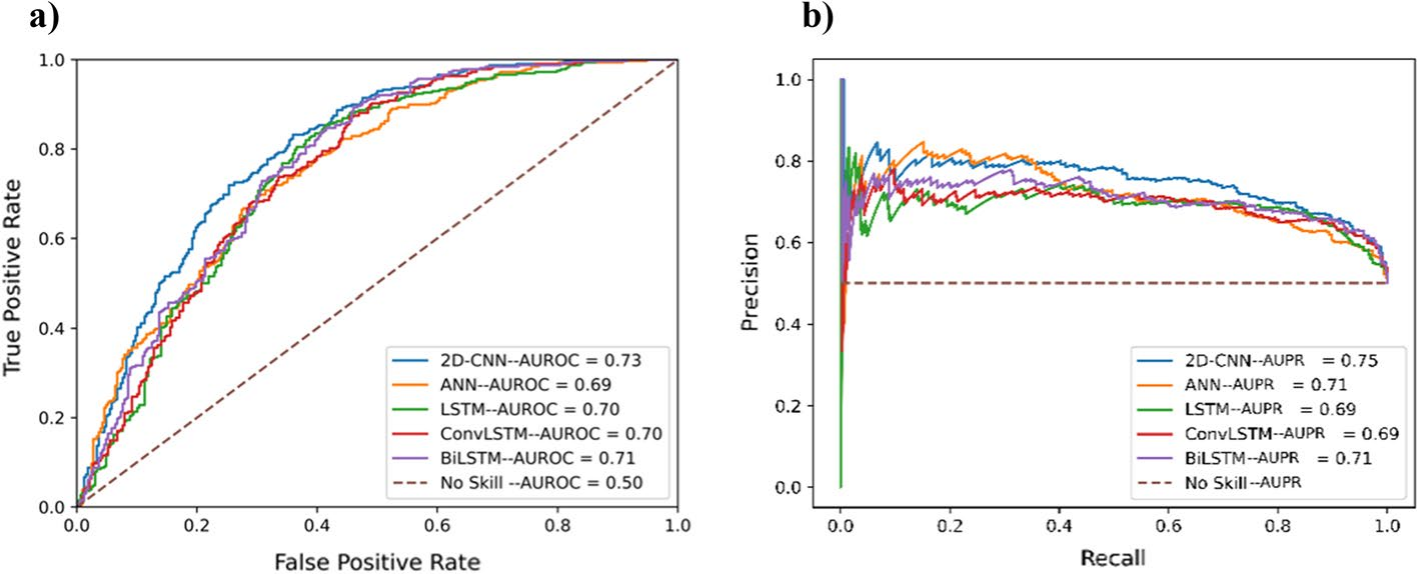
**a** ROC curves and area under curve (AUC) values for different architectures for the supervised embedding layer module. **b** Precision-recall (PR) curves and area under curve (AUC) values for different architectures for the supervised embedding layer module

To obtain the best architecture for the word embedding module, we performed a fivefold cross-validation of the model using various architectures: 2D-CNN [29], ANN, LSTM [30], ConvLSTM [31], and BiLSTM using the value of window size (= 37), vocabulary size (= 23) and embedding dimension (= 4) obtained from the prior experiments. It must be noted here that the supervised word embedding is obtained as a part of the training process of the model, so we only experimented with DL-based architectures. These DL architectures were tuned using grid search with fivefold cross-validation over wide range of search space (provided in Additional file 1: Table S2). The results of the fivefold cross-validation of the optimized models are shown in Table 3. Similarly, the AUPR and AUC for cross-validation for these models are shown in Fig. 4. It can be observed from Table 3 as well as Fig. 4 that the 2D-CNN architecture produces the best results (MCC in the table and AUC in the figures). Based on this, 2D-CNN architecture was chosen as the final architecture for the word embedding module.

**Table 3:** Fivefold cross-validation results (mean ± one standard deviation) of embedding layer module on the training set

Model	ACC	SN	SP	MCC
2D-CNN	**0.688** ± **0.018**	0.760 ± 0.063	0.615 ± 0.069	**0.382** ± **0.034**
ANN	0.658 ± 0.018	0.697 ± 0.0351	0.619 ± 0.010	0.318 ± 0.036
LSTM	0.674 ± 0.011	**0.816** ± **0.067**	0.533 ± 0.074	0.368 ± 0.024
ConvLSTM	0.667 ± 0.006	0.836 ± 0.023	0.498 ± 0.017	0.355 ± 0.017
BiLSTM	0.686 ± 0.009	0.747 ± 0.093	**0.626** ± **0.083**	0.380 ± 0.022

The highest values in each category are bolded

### Selection of model architecture for the pLM module (protT5)

**Fig. 5:**
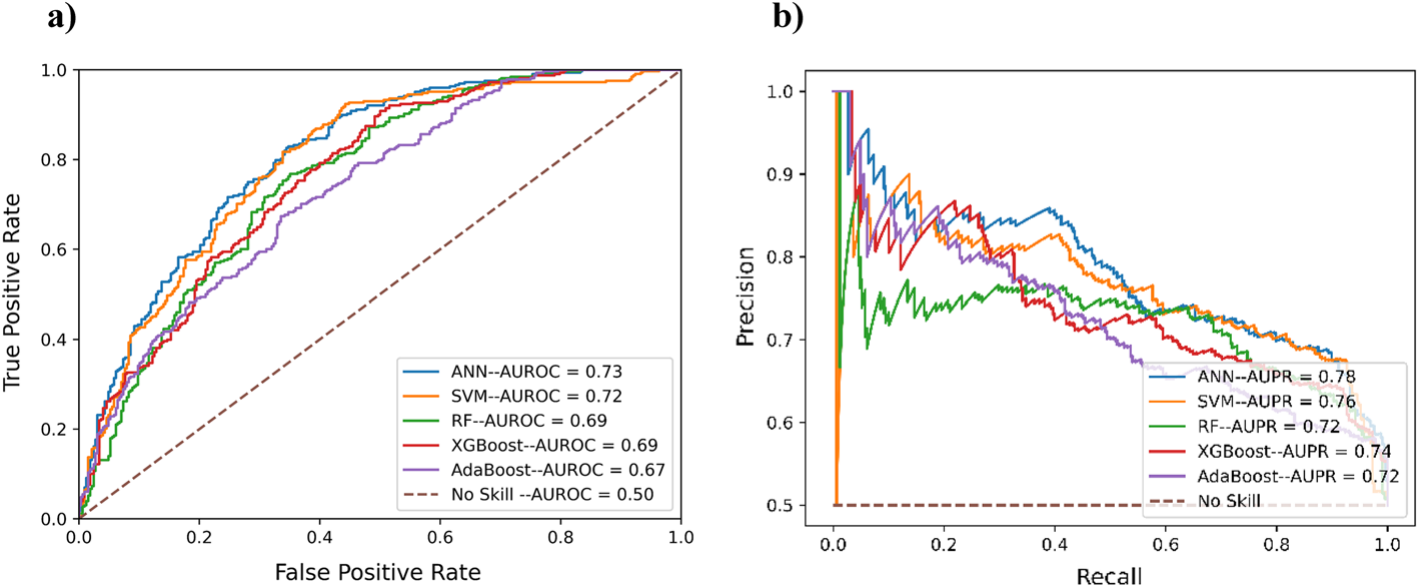
**a** Area under ROC curves (AUROC) values for different architectures for the ProtT5 module. **b** Area under precision-recall (AUPR) values for different architectures for the ProtT5 module

It has been observed in multiple studies encompassing various bioinformatics tasks that a simple machine learning model is enough to obtain a satisfactory performance for pLM based embeddings [26, 27]. Based on this knowledge, we experimented with ANN (Artificial Neural Network), SVM (Support Vector Machine) [35], RF (Random Forest) [36], XGBoost (Extreme Gradient Boosting), and AdaBoost (Adaptive Boosting) architectures for protT5 module using fivefold cross-validation. The scikit-learn’s GridsearchCV was used to optimize SVM, RF, XGBoost and AdaBoost with *cv* as 5 and *param_grid* (parameters grid) value as mentioned in the Additional file 1: Table S1. The results of the fivefold cross-validation of the optimal models are reported in Table 3 and ROC and PR curves for the same are shown in Fig. 5. It can be observed that the ANN architecture produced the best results (MCC in Table 4 and AUC in Fig. 5). Based on this, ANN architecture was chosen as the final architecture for the pLM ProtT5 module.

**Table 4:** Fivefold cross validation results (mean ± one standard deviation) of different models based on ProtT5 features

Architecture	ACC	SN	SP	MCC
ANN	**0.710 ± 0.015**	0.745 ± 0.028	0.674 ± 0.015	**0.421** ± **0.030**
SVM	0.700 ± 0.012	0.702 ± 0.016	**0.699 ± 0.020**	0.401 ± 0.024
RF	0.682 ± 0.010	**0.815 ± 0.815**	0.549 ± 0.815	0.379 ± 0.378
XGBoost	0.699 ± 0.008	0.752 ± 0.019	0.645 ± 0.007	0.400 ± 0.016
AdaBoost	0.672 ± 0143	0.695 ± 0.024	0.650 ± 0.022	0.345 ± 0.029

Highest values in each column are highlighted in bold

### Selection of model architecture for meta classifier

**Fig. 6:**
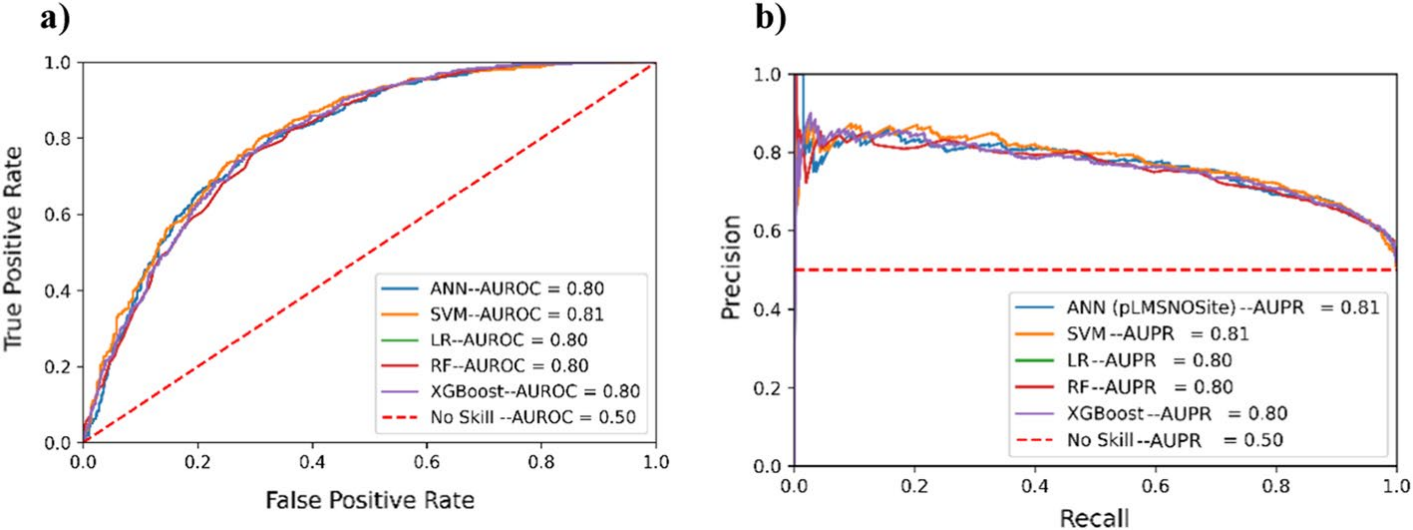
Results based on fivefold cross-validation **a** ROC curves and area under curve (AUC) values for different architectures for the meta classifier model. **b** Precision-recall (PR) curves and area under curve (AUC) values for different architectures for meta classifier model

Additionally, the optimal architecture for the stacked generalization (aka meta classifier) was obtained using fivefold cross-validation on various ML models. Essentially, during the cross-validation of models for the meta-classifier, the intermediate features obtained from base models were used paying special attention to any potential leakage of target information in the training of the meta classifier as described in the methods section. The candidate models for the meta-classifier were optimized using this approach (data leakage mitigation) for fivefold cross-validation (over the search space reported in Additional file 1: Table S3). Table 5 and Fig. 6 show the comparison of the optimized models based on fivefold cross-validation. These results indicate that Artificial Neural networks (ANN) achieves better validation performance compared to other classifiers in terms of MCC and competitive results in terms of AUPR and AUROC. The meta-classifier based on ANN was hence chosen for our work and we call the overall approach as pLMSNOSite.

**Table 5:** Performance comparison using different architectures for meta-classifier based on fivefold cross-validation results (mean ± one standard deviation)

Model	ACC	SN	SP	MCC
ANN	**0.727 ± 0.017**	0.769 ± 0.016	**0.685 ± 0.033**	**0.4573 ± 0.032**
LR	0.703 ± 0.014	0.740 ± 0.017	0.665 ± 0.028	0.407 ± 0.027
SVM	0.719 ± 0.021	**0.807 ± 0.029**	0.631 ± 0.017	0.445 ± 0.043
RF	0.724 ± 0.010	0.771 ± 0.026	0.678 ± 0.022	0.451 ± 0.021
XGBoost	0.697 ± 0.006	0.735 ± 0.014	0.660 ± 0.022	0.396 ± 0.011

The highest value in each column is highlighted in bold

### Performance of base models and pLMSNOSite on independent test set

**Fig. 7:**
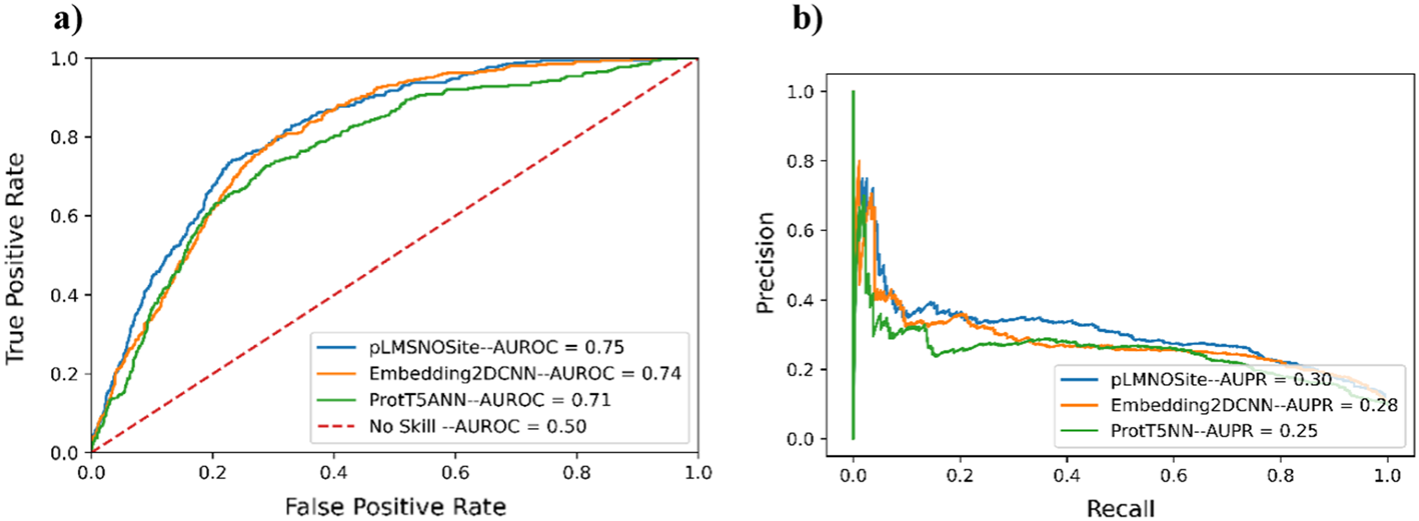
Results based on independent test set (imbalanced): **a** ROC curve and **b** AUPR curve for the base models and pLMSNOSite

To observe the relative performance of the base models (aka Embedding2DCNN and ProtT5ANN) and ensemble model on the independent test set, we compared the performance of these models using an independent test set. Note that this independent test set is imbalanced and that these results have no effect whatsoever on model selection (model selection was solely done based on the results of fivefold cross-validation on training data). The ROC and PR curves of the base models and ensemble model (pLMSNOSite) are shown in Fig. 7 and Table 6 shows other performance metrics for the base models and ensemble model. The results indicate that the ensemble model (pLMSNOSite) exhibits higher AUROC, AUPR and MCC compared to the base models. This demonstrates the better generalization ability of the ensemble model (pLMSNOSite) compared to the base models. From the figure, the AUPR values are quite low which is to be expected because precision and recall are focused on minority class (minority class size: 351, majority class size: 3168). Nevertheless, pLMSNOSite still has better precision compared to other existing approaches (Additional file 1: Fig. S4).

**Table 6:** Performance comparison of base models (aka Embedding2DCNN and ProtT5ANN models) and ensemble model (pLMSNOSite)

Models	ACC	SN	SP	MCC
Embedding2DCNN	0.706	**0.798**	0.696	0.310
ProtT5ANN	**0.791**	0.598	**0.812**	0.293
pLMSNOSite	0.769	0.735	0.772	**0.340**

The highest value in each column is highlighted in bold

**Fig. 8:**
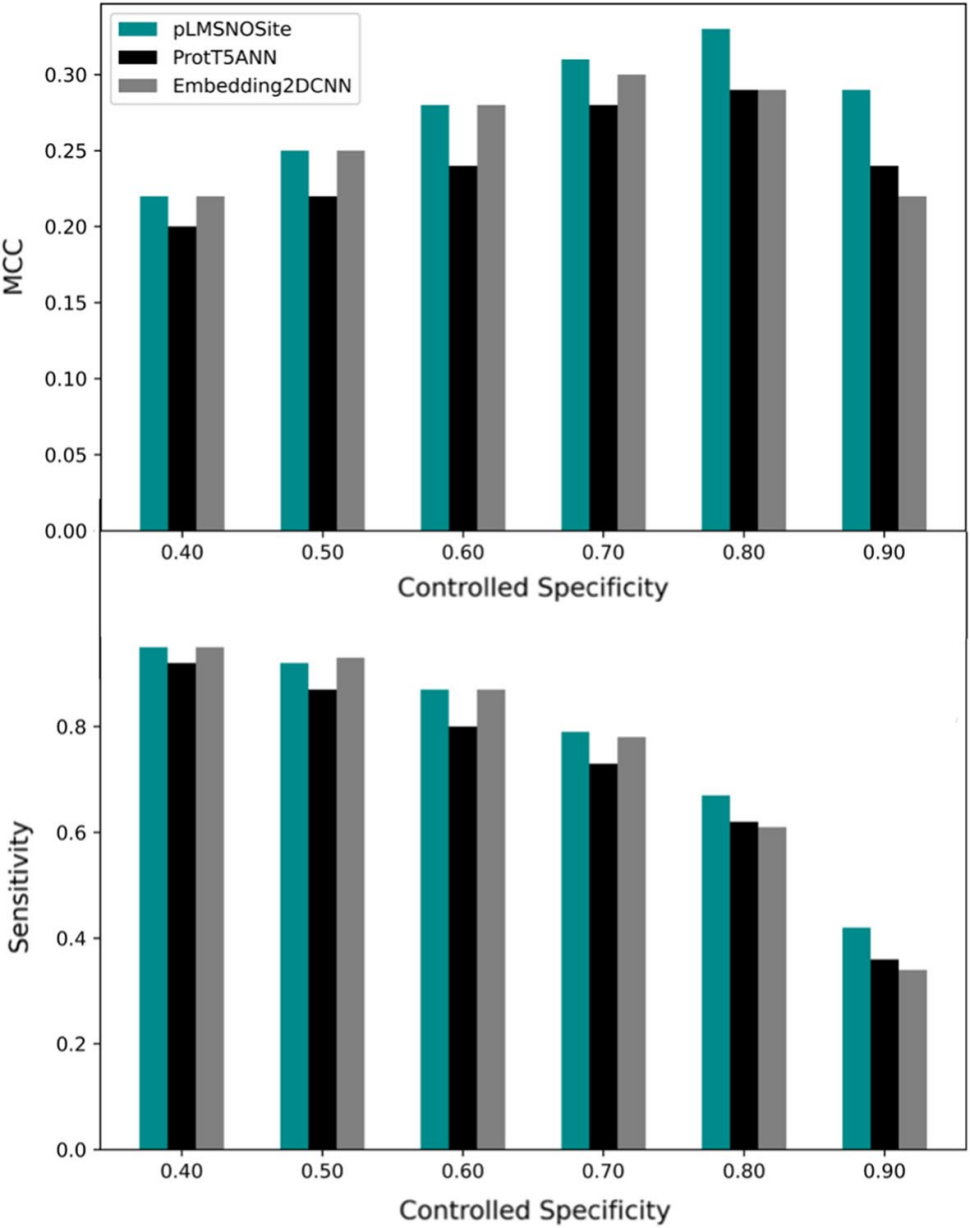
Comparison of MCC and Sensitivity of pLMSNOSite with base models under different controlled specificity values

Furthermore, we analyzed the performance of pLMSNOSite and base models under various controlled specificity values. As shown in Fig. 8, we can observe that the proposed pLMSNOSite approach performs better in terms of MCC and sensitivity at various values of controlled specificity. Also, we can concur that as the models become more specific, pLMSNOSite is still able to outperform the base models.

It must be noted that pLMSNOSite was selected as the final predictor based on the cross-validation experiments, and these results were presented to simply assess the performance of the base models and meta-model on the independent test set.

### Comparison with other existing tools using an independent test set

Finally, we compared the performance of our approach (pLMNOSite) with other existing SNO site prediction tools using an independent test set described in the Benchmark dataset section. Specifically, our approach was compared against widely available tools such as GPS-SNO [9], SNOSite [10], iSNO-PseAAC [11], DeepNitro [15], and PreSNO [16]. It must be pointed out that the same training and independent test set used by PreSNO predictor was employed for our analysis for fair comparison. The results of the comparison are presented in Table 7 and note that the results for other predictors were adopted from PreSNO [16]. It can be observed from Table 7 that the pLMSNOSite achieves the best MCC (= 0.340) among the compared approaches showing an improvement of ∼35.0% in MCC compared to the next best approach (PreSNO). Additionally, it also exhibited an ∼21.7% increase in sensitivity and improvements in terms of specificity and accuracy. It is worth noting that pLMNOSite struck the most balance between sensitivity and specificity with a g-mean (geometric mean of sensitivity and specificity) of 0.754, a ∼10.6% improvement over PreSNO. Additionally, it can also be seen that the ProtT5 model alone has a better MCC (= 0.293) than the other compared approaches. Based on these results, it can be concluded that our novel approach termed pLMSNOSite is a robust predictor of S-nitrosylation sites in proteins.

**Table 7:** Performance comparison of pLMSNOSite against other existing approaches using the independent test set

Predictors	TP	FP	TN	FN	ACC	SN	SP	MCC	AUROC
GPS-SNO	99	825	2337	253	0.693	0.281	0.739	0.014	0.523
iSNO-PseAAC	101	768	2394	251	0.710	0.287	0.757	0.031	–
SNOSite	235	1749	1413	117	0.469	0.668	0.447	0.069	–
DeepNitro	202	776	2386	148	0.737	0.578	0.737	0.222	0.731
PreSNO	211	733	2431	141	0.752	0.604	0.769	0.252	**0.756**
pLMSNOSite	**258**	**718**	**2446**	**93**	**0.769**	**0.735**	**0.773**	**0.340**	0.754

The highest values in each column are highlighted in bold

Note that the values for other approaches were adopted from PreSNO. Although same independent test set was used for all the approaches, there is a slight variation in the number of total positive and negative sites. Nevertheless, the integrity of comparison is not compromised at all

### t-SNE visualization of pLMSNOSite

**Fig. 9:**
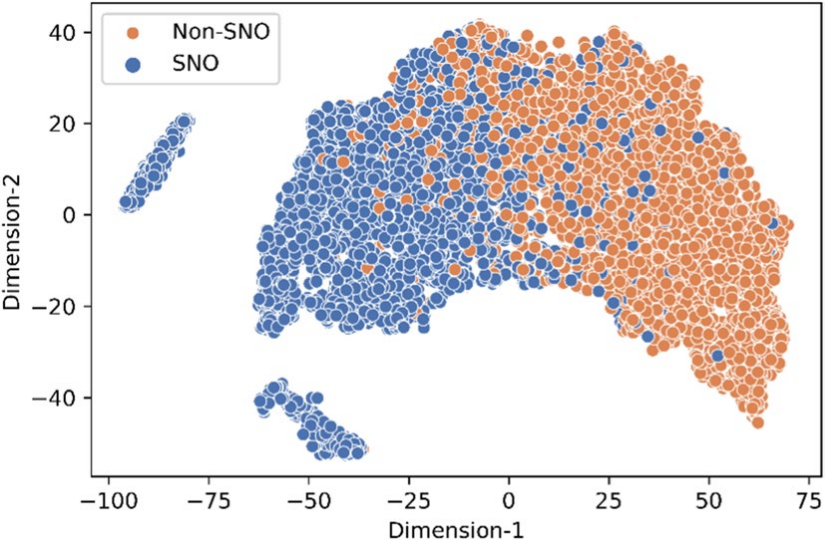
2D t-SNE visualization of the learned features from training data by pLMSNOSite

Additionally, we used t-distributed stochastic neighbor embedding (t-SNE) [37] to project the learned features from the final hidden layer into R^2^ cartesian space. With a perplexity value of 50 and a learning rate of 500, the t-SNE was visualized from the training data using a scatter plot (Fig. 9). It can be inferred from the plot that the boundary of separation between SNO sites (blue data points) and non-SNO sites (orange data points) is quite pronounced indicating that the proposed stacked generalization approach is able to discriminate between the positive sites and the negative sites.

## Discussions and conclusions

Protein S-nitrosylation is one of the important protein post-translational modifications that is responsible for regulating protein functions and cell signaling of all main classes of proteins. In this work, we developed a computational tool to predict protein S-nitrosylation sites called pLMSNOSite that combines a supervised embedding layer model and a protein language model (based on ProtT5) using a stacked generalization approach. Based on independent test results, pLMSNOSite shows better performance than the compared existing tools. As can be seen from the results, the improved performance of our approach can mainly be attributed to the new embedding representation obtained from ProtT5 (a protein language model). One of the benefits of language models like ProtT5 is that it is learned on overall sequence to extract the contextualized embedding of the site of interest as a consequence of which the dependency on defining local contextual information of the site based on the window size (which demands additional overhead for hyperparameter tuning) is averted.  Based on the experimental results, it can be concluded that pLMSNOSite is a promising tool for predicting protein S-nitrosylation sites. The trained pLMSNOSite model and related dataset are provided in our public GitHub repository (https://github.com/KCLabMTU/pLMSNOSite) for the community.

As in pLMSNOSite, the representation of protein sequences using protein language model could be explored to improve other protein bioinformatics tasks like protein-drug interaction prediction [38]. Essentially, by representing the protein target using pLMs we may expect improved protein-drug interaction prediction. Additionally, the protein language model could be used for improved protein–protein interaction prediction (PPI) [39] where representations for both proteins can be extracted using pLMs. Although pLMSNOSite shows promising performance, the predictive performance of pLMSNOSite could be improved by leveraging the vast amount of structural data made available due to the success of AlphaFold2 [18]. Additionally, pLMSNOSite only uses sequence features from ProtT5 language model for feature extraction but there are other recent protein language models (e.g. ESM-2 [24]) and exploration of these language models for SNO site prediction could be other important future work. Since our method uses ProtT5, our method might require appropriate computational resources for very long protein sequences.

## Supplementary Information

**Additional file 1.** Contains supplementary tables and figures referred to in the manuscript. In sections 1, 2, and 3, we describe various ML/DL architectures and their respective hyperparameters. **Table S1**. Hyperparameter search space for models in the ProtT5 module. **Table S2**. Hyperparameter search space for models in the word embedding module. **Table S3**. Hyperparameter search space for models in the meta-classifier. **Table S4**. fivefold cross-validation results of Embedding2DCNN and ProtT5ANN when imbalanced learning (based on cost-sensitive learning) is performed. **Table S5**. Best combination (with respect to MCC) of window size and embedding dimension for each of the candidate models for the word embedding module on fivefold cross-validation. **Figure S1**. The sensitive analysis curves of each DL model in the word embedding module on fivefold cross-validation. **Table S6**. Comparison of ProtT5 with other pLMs such as ProtBERT (BERT-based ProtTrans family model) and Meta’s ESM-1 using independent testing. **Figure S2**. Frequency and WebLogo plots for train positive and train negative window sequences (window size = 37). **Figure S3**. Precision-Recall curves were produced for base models and pLMSNOSite using an imbalanced independent set and a balanced independent test set separately. **Figure S4**. Comparison of pLMSNOSite with other existing predictors based on precision values using an independent test set.

## Data Availability

The datasets, trained models, source codes, and other resources used in this study are publicly available https://github.com/KCLabMTU/pLMSNOSite.

## Abbreviations

SNO: S-nitrosylation
LMs: Language models
T5: Text to text transfer transformer
t-SNE: T-distributed stochastic neighbor embedding
PTM: Post translational modification
MCC: Mathew correlation coefficient
ROC: Receiver operating characteristics
AUC: Area under ROC curve
PR: Precision-recall
ReLU: Rectified linear unit
CNN: Convolutional neural network
LSTM: Long short-term memory
BiLSTM: Bidirectional long short-term memory
ConvLSTM: Convolutional long short-term memory
DL: Deep learning
SVM: Support vector machine
LR: Logistic regression
RF: Random forest
PPI: Protein–protein interaction
