# Supplementary material for: pLMSNOSite: an ensemble-based approach for predicting protein S-nitrosylation sites by integrating supervised word embedding and embedding from pre-trained protein language model
Source: BMC Bioinformatics. 2023 Feb 8;24:41. doi: 10.1186/s12859-023-05164-9 (PMC9909867; doi:10.1186/s12859-023-05164-9)

**Supplementary Materials for:**

**pLMSNOSite: an ensemble-based approach for predicting protein S-nitrosylation sites by integrating supervised word embedding and embedding from pre-trained protein language model**

## Pawel Pratyush^1^, Suresh Pokharel^1^, Hiroto Saigo^2^, Dukka B KC^1*^

1. Department of Computer Science, Michigan Technological University, Houghton, MI, USA.

2. Department of Electrical Engineering and Computer Science, Kyushu University, 744, Motooka, Nishi-ku, 819-0395, Japan

* Corresponding Author: dbkc@mtu.edu

**1.Different architectures for supervised word embedding module**

Following deep learning architectures were explored for the supervised word embedding module. These architectures and their hyperparameters are optimized using 5-fold cross-validation . All the architectures were implemented in Keras library using the Tensorflow backend.

**Architecture 1 : Artificial Neural Network(ANN) architecture:** The model contains 5 layers - Embedding Layer, Lambda Layer, Flatten Layer, a fully connected later and a output layer. The loss function used is binary cross entropy and optimizer is Adam.

Input Layer: The protein sequences of window size 37 were integer encoded with the feature space of 23(added ‘-‘ for no sequence) providing a shape of (37,23).

Embedding Layer: Dimension - 23 x 4 x 37 (23 is vocabulary size, 4 is the embedding dimension and 37 is the window size or input length). Lambda layer and flatten layer follows this layer.

Fully connected layer: Contains a single hidden layer with 8 neurons with ReLU activation.

Output layer: It uses sigmoid activation.

**Architecture 2: LSTM Architecture:** The model contains of 6 layers with 2 hidden layers. The loss function used is binary cross entropy and the used optimizer is Adam.

Input Layer: The protein sequences of window size 37 were integer encoded with the feature space of 23(added ‘-‘ for no sequence) providing a shape of (37,23).

Embedding Layer: Dimension - 23 x 4 x 37 (23 is vocabulary size, 4 is the embedding dimension and 37 is the window size or input length).

LSTM Layer: Contains 32 units, input shape of 37 x 23 as (timesteps, features) with stateful kept as False. Flatten Layer follows this layer.

Fully connected layer: 2 hidden layers with 32 and 4 neurons respectively each with ReLU activations.

Output layer: It uses sigmoid activation.

**Architecture 3: Conv-LSTM Architecture:** The model contains 6 layers. The loss function used is binary cross entropy and the used optimizer is Adam.

Input Layer: The protein sequences of window size 37 were integer encoded with the feature space of 23(added ‘-‘ for no sequence) providing a shape of (37,23).

Embedding Layer: Dimension - 23 x 4 x 37 (23 is vocabulary size, 4 is the embedding dimension and 37 is the window size or input length. Lambda layer follows this layer.

ConvLSTM Layer: ConvLSTM1D with Number of Filters= 64 and kernel_size=4. Flatten layer follows this layer.

Fully connected layer: A hidden layer with 16 neurons having ReLU activation.

Output layer: It uses sigmoid activation and generates singe output in the range [0,1].

**Architecture 4: BiLSTM Architecture:** The model contains 5 layers. The loss function used is binary cross entropy and the used optimizer is Adam.

Input Layer: The protein sequences of window size 37 were integer encoded with the feature space of 23(added ‘-‘ for no sequence) providing a shape of (37,23).

Embedding Layer: Dimension - 23 x 4 x 37 (23 is vocabulary size, 4 is the embedding dimension and 37 is the window size or input length).

BiLSTM Layer: with LSTM having 16 units. Flatten layer follows this layer.

Fully connected layer: A hidden layer with 32 neurons having ReLU activation.

Output layer: It uses sigmoid activation.

**Architecture 5: CNN-2D Architecture (Embedding2DCNN):** The model contains 8 layers. The loss function used is binary cross entropy and the used optimizer is Adam.

Input Layer: The protein sequences of window size 37 were integer encoded with the feature space of 23(added ‘-‘ for no sequence) providing a shape of (37,23).

Embedding Layer: Dimension - 23 x 4 x 37 (23 is vocabulary size, 4 is the embedding dimension and 37 is the window size or input length). Lambda layer follows this layer.

Conv2D Layer: 64 filters, kernel_size=(19,1), activation=ReLU.

Dropout layer: 0.3

MaxPooling2D layer: pool_size=(5,2)

Flatten Layer

Fully connected layer: A hidden layer with 16 neurons having ReLU activation.

Output layer: It uses sigmoid activation.

**2. Different ML/DL architectures for ProtT5 module**

Following ML/DL architectures were explored for the ProtT5 module. The hyperparameters were determined with grid search on 5-fold cross validation using scikit-learn’s GridSearchCV.

**Support Vector Machine**: regularization parameter C = 7

**Random Forest**: max_depth=20 and max_features= ‘auto’

**XGBoost**: ‘gamma’ = 0 and max_depth = 6

**AdaBoost**: ‘n-estimators’=100

**ANN(ProtT5ANN):** Consist of 7 layers each with ReLU activation and the optimizer used is Adam and binary cross entropy loss.

Input Layer – 1024 feature vector from protT5 pLM.

Dense Layer having 128 neurons

Dropout layer with 0.4

Dense Layer having 16 neurons

Dropout Layer with 0.2

Dense Layer having 4 neurons

Output layer with sigmoid activation

**3. Architecture for the meta-classifier**

The following ML/DL architectures were explored for the meta-classifier: ANN, LR, SVM, RF, and XGBOOST.

**4. Hyperparameter Search Space for each module**

**Supplementary Table 1:** **Search space for ProtT5 module:**

| **Models** | **Hyperparameter** |
| --- | --- |
| ANN | Number of layer - {1,2,3}  Number of neurons - {16,32,64,128,256,512} |
| SVM | C - {1,2,3,4,5,6,7,8,9,10}  kernel- {linear,rbf} |
| RF | max depth - {10,20,30,40,50,60,70,80}  max- features - {‘auto’,’sqrt’} |
| XGBoost | ‘max-depth’- {10,20,30,40,50,60,70,80}  ‘gamma’ - {0,0.5, 1, 1.5, 2, 5} |
| AdaBoost | ‘number of estimators’ - {10, 50, 100, 500} |

**Supplementary Table 2:** **Search space for supervised word embedding module**

| **Models** | **Parameters** |
| --- | --- |
| ANN | Number of layer - 1,2,3  Number of neurons - 16,32,64,128,256,512  Batch size - 64,128,256 |
| CNN | No of filters - {16,32,64,128,256}  No of CNN layers - 1,2,3  No of fully connected layers- 1,2  No of neurons in fully connected layers - 8,32,64,128,256 |
| LSTM | LSTM layers - {1,2}  Units -{16,32,64}  No of fully connected layers- 1,2  No of neurons in fully connected layers - 8,32,64,128,256 |
| Convlstm | No of Convlstm layers- {1,2}  No of filters: {16,32,64,128}  No of fully connected layers- 1,2  No of neurons in fully connected layers - 8,32,64,128,256 |
| BiLSTM | no. of BiLSTM layers: {1,2}  No. of units: {4,16,32,64}  No of fully connected layers- 1,2  No of neurons in fully connected layers - 8,32,64,128,256 |

**Supplementary Table 3:** **Search space for meta-classifier**

| **Models** | **Hyperparameter** |
| --- | --- |
| ANN | Number of layer - 1,2,3  Number of neurons - 16,32,64,128,256,512  Batch size - 64,128,256 |
| LR | var_smoothing - np.logspace(0,-9, num=100) |
| SVM | C - {1,2,3,4,5,6,7,8,9,10}  kernel- {linear,rbf} |
| RF | max depth - {10,20,30,40,50,60,70,80}  max- features - {‘auto’,’sqrt’} |
| XGBoost | ‘max-depth’- {10,20,30,40,50,60,70,80}  ‘gamma’ - {0,0.5, 1, 1.5, 2, 5} |

**5. Imbalanced Learning Result**

We also performed the imbalance learning using the cost-sensitive method where the weights for each class were assigned using the following formula:

*class_weight = n_samples / (n_classes * n_samples_with_class)*

where *n_samples* = total number of samples

*n_classes* = total number of class (in this case =2)

*n_samples _with_class* = the number of samples in the class

The following table shows the results obtained from 5-fold cross-validation on word embedding features and protT5 features using cost-sensitive learning.

**Supplementary Table 4:** 5-fold cross-validation results (mean ± standard deviation) when imbalanced learning is performed on Embedding2DCNN (word embedding) and ProtT5ANN (ProtT5)

| **Module** | **Sensitivity** | **Specificity** | **MCC** |
| --- | --- | --- | --- |
| Embedding2DCNN | 0.485+0.121 | 0.764+0.172 | 0.209+0.078 |
| ProtT5ANN | 0.539+ 0.042 | 0.757+0.017 | 0.243 + 0.023 |

As seen from the table the results from imbalance learning were not as good as balanced learning, thus we decided to use the balanced learning. One thing to note here is that the negative sites might not all be true negatives, and this is the reason why we believe we did not get good results when using all the negative sites (imbalance learning).

**6. Selection of Window size and Embedding Dimension**

Window size and embedding dimension are parameters in the word embedding module. Essentially, we did a grid search based on MCC on window size values ranging from 21 to 63 and embedding dimension values ranging from 4 to 32. The best combination was hence determined for each model architecture. The 2D-CNN produced the highest MCC followed by BiLSTM. Hence, the window size of 37 and the embedding dimension of 4 were fixed for further analysis based on 2D-CNN architecture.

**Supplementary Table 5:** Best combination of window size and embedding dimension on 5-fold cross-validation for each candidate models for word embedding module

| Models | Maximum MCC | Window (input_length) | Embedding dimension(output_dim) |
| --- | --- | --- | --- |
| 2D-CNN | **0.382** | 37 | 4 |
| ANN | 0.328 | 41 | 6 |
| LSTM | 0.376 | 49 | 10 |
| ConvLSTM | 0.378 | 47 | 11 |
| BiLSTM | 0.381 | 37 | 10 |

The window size for the two best performing models (2D-CNN and BiLSTM) is 37 and hence window size of 37 are fixed. Interestingly, the previous work PreSNO found the optimal window size as 41 which is slightly more than our work (only 2 more residues on each side of the central residue). Consequently, we select the embedding dimension as 4 with respect to 2D-CNN. These values were fixed for carrying out further analysis.

**Supplementary Figure 1:** The sensitivity analysis of each individual models by fixing their respective optimal window size and optimal embedding dimension is presented below:

| **Models** | **Fixing embedding dimension and vocab size (=23) while varying window size** | **Fixing window size and vocab size (=23) while varying embedding dimension** |
| --- | --- | --- |
| 2D-CNN | 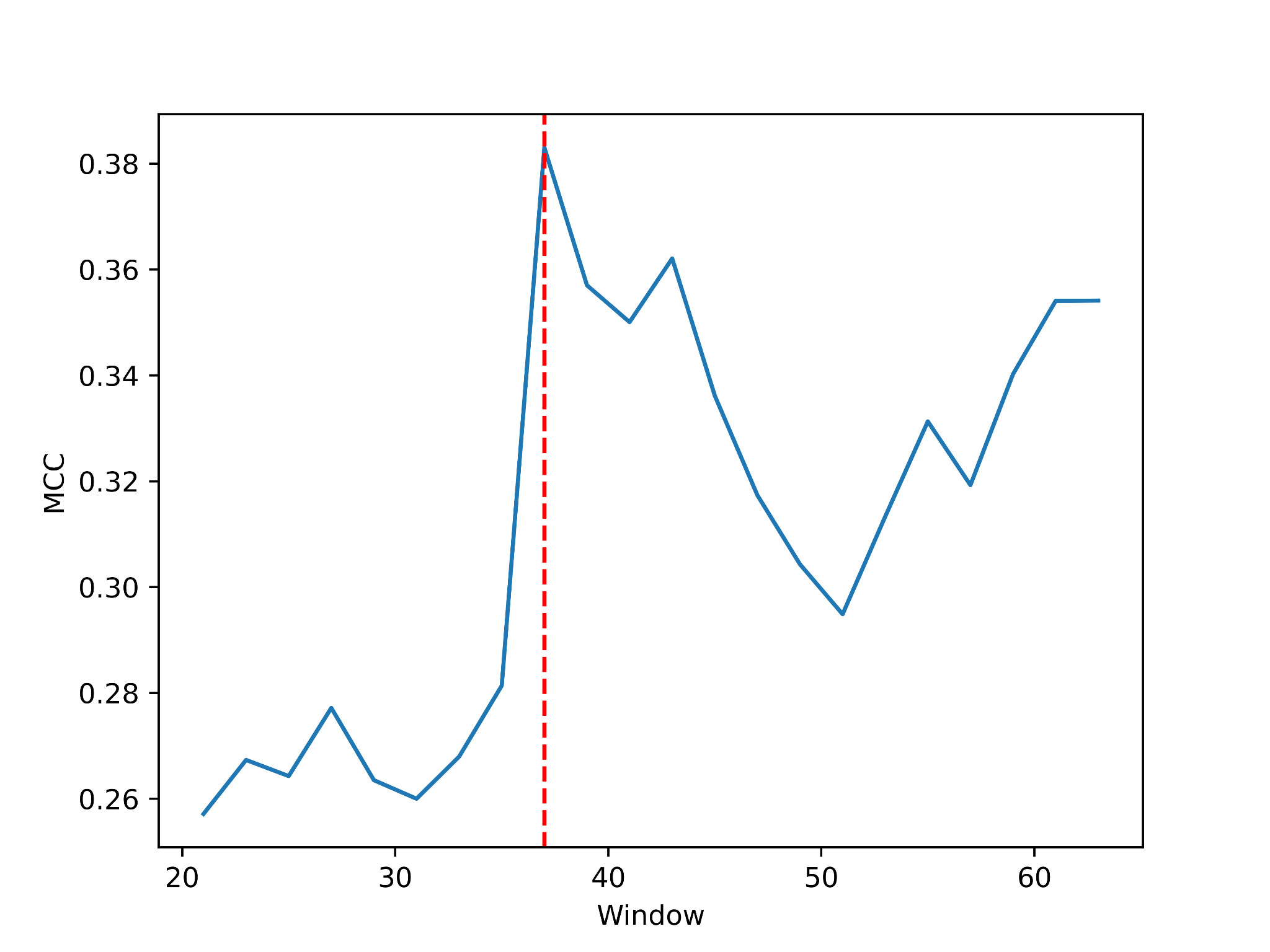  Embedding Dimension = 4, vocab size =23 | 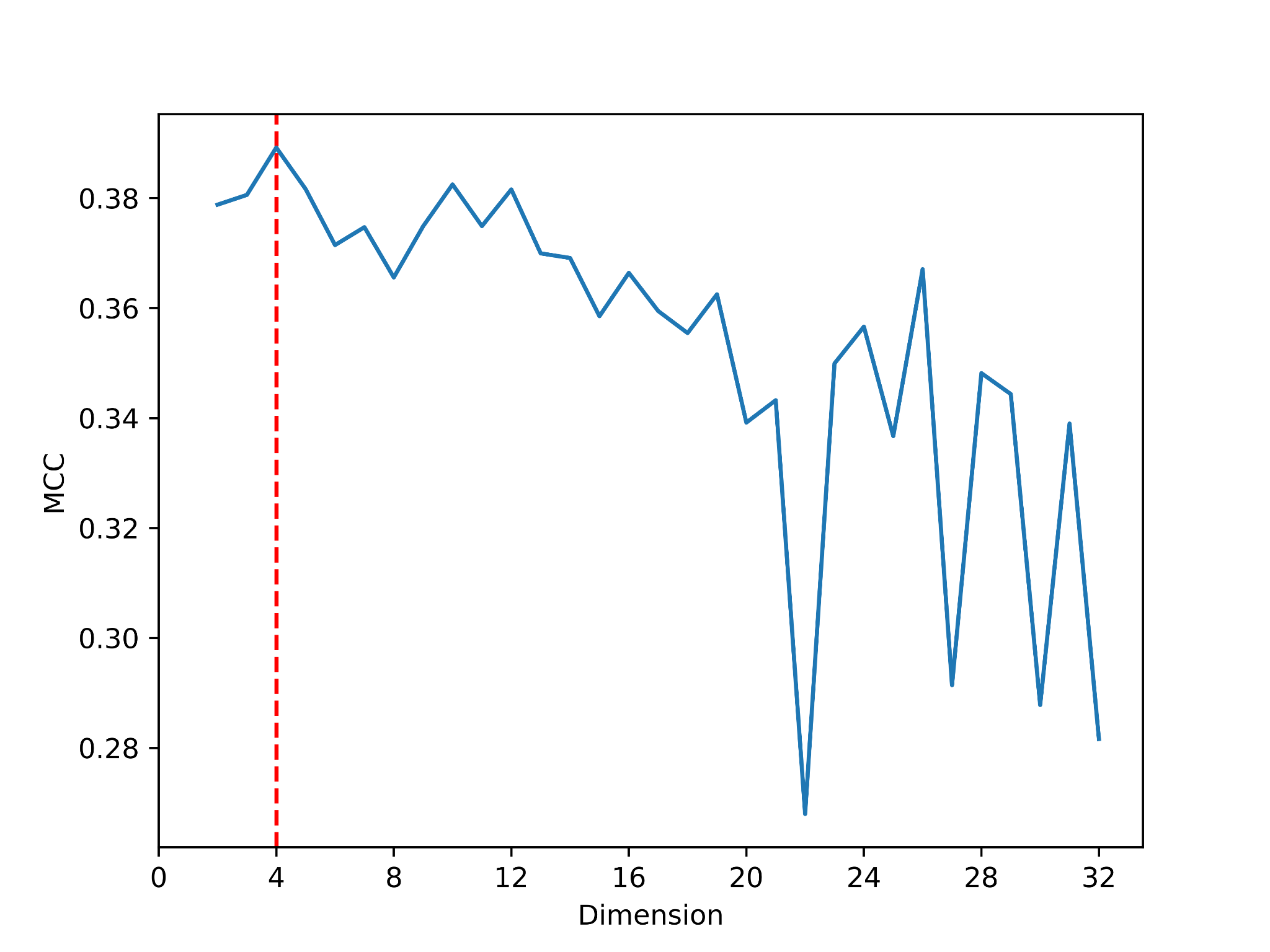  window size = 37, vocab size =23 |
| ANN | 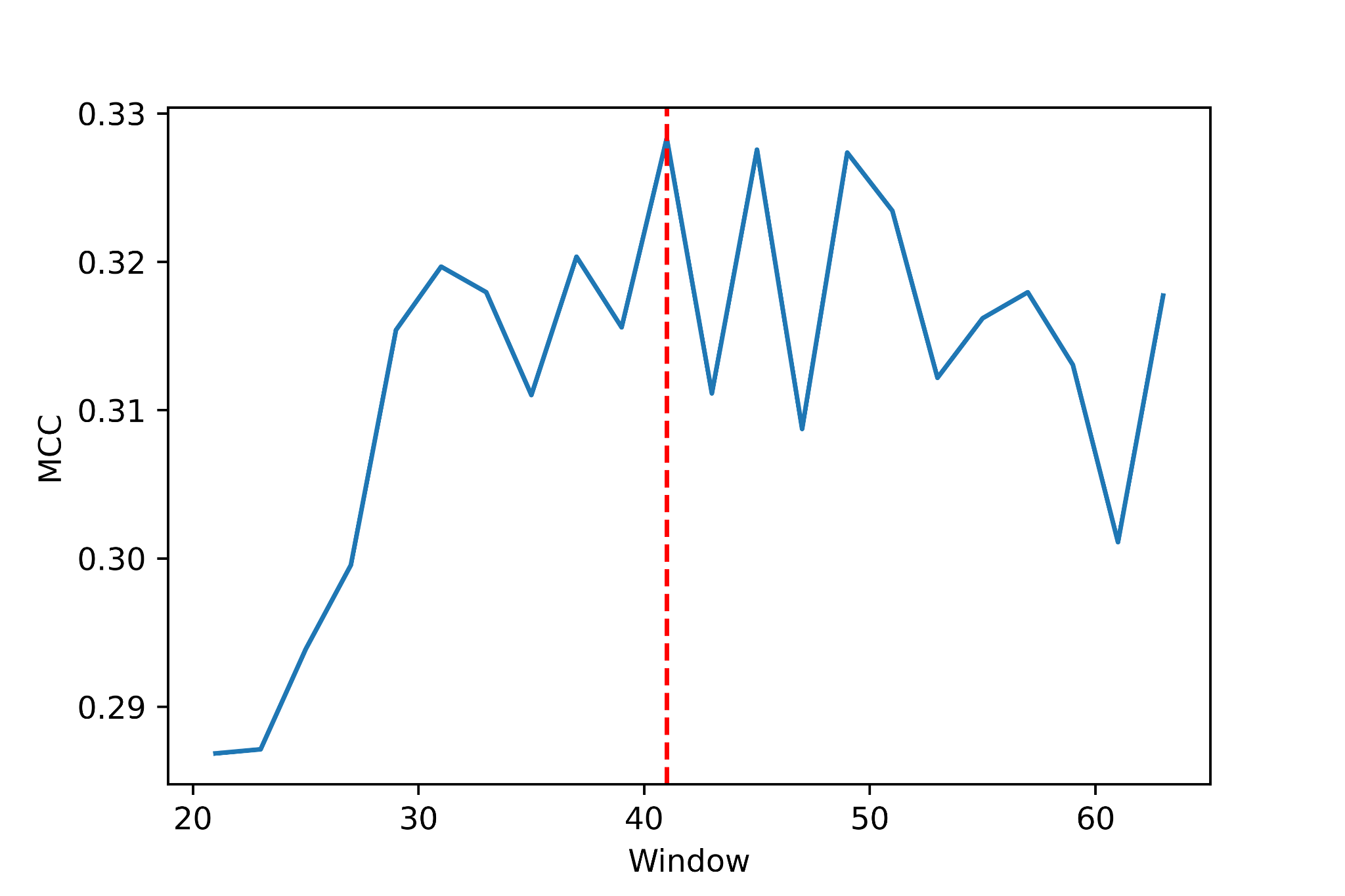  Embedding Dimension = 6, vocab size =23 | 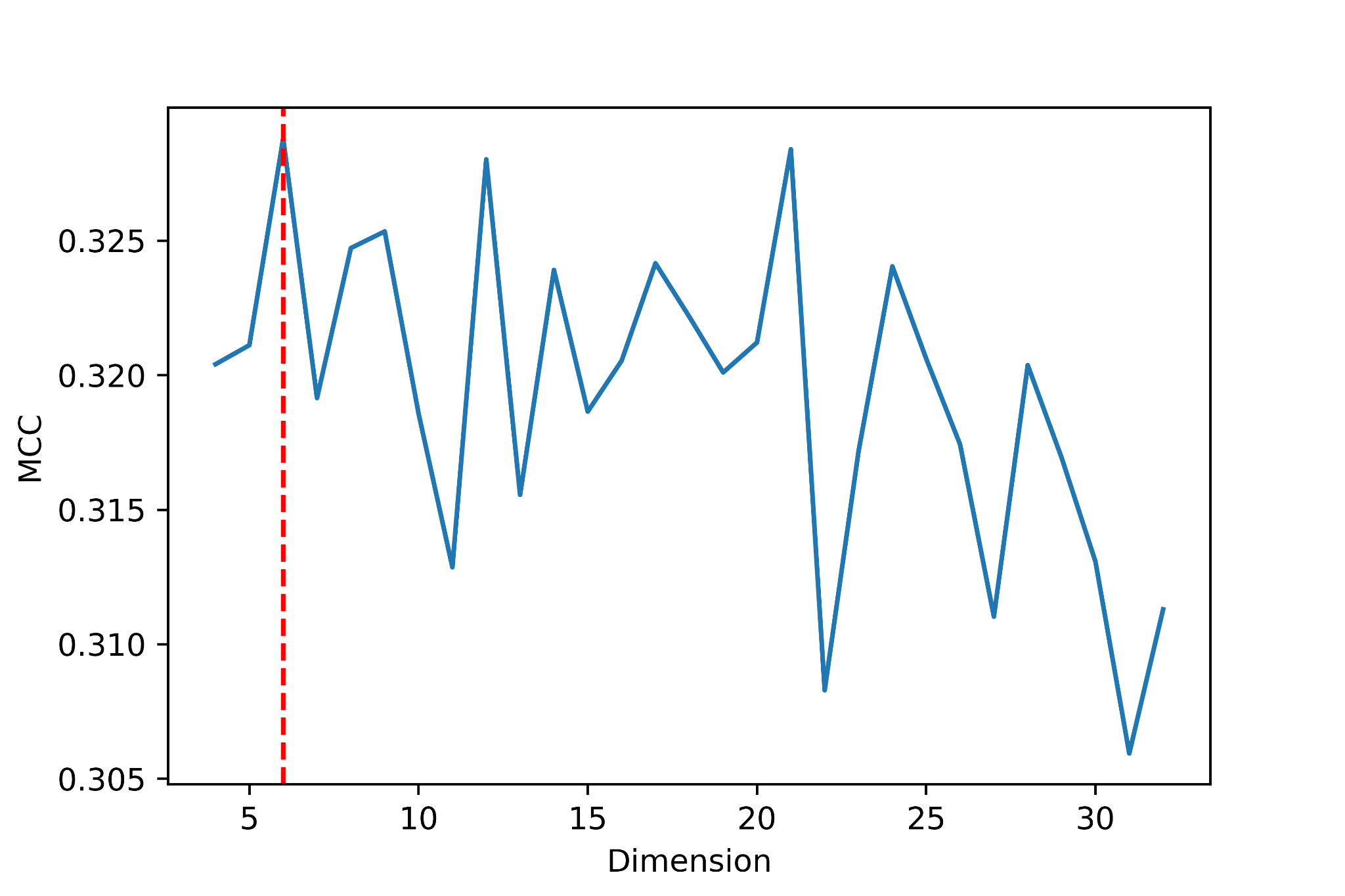  window size = 41, vocab size =23 |
| LSTM | 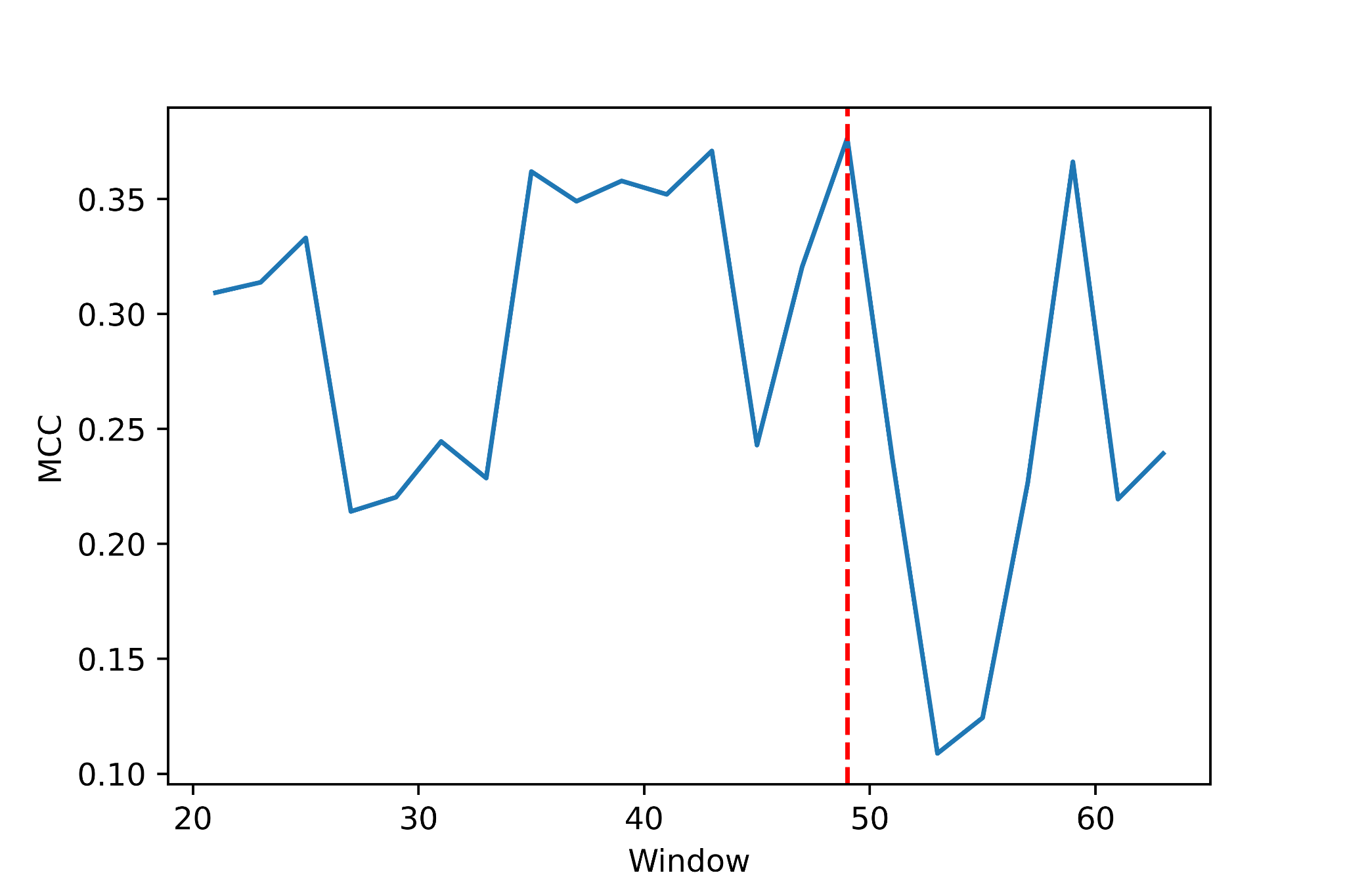  Embbeding Dimension = 10, vocab size 23 | 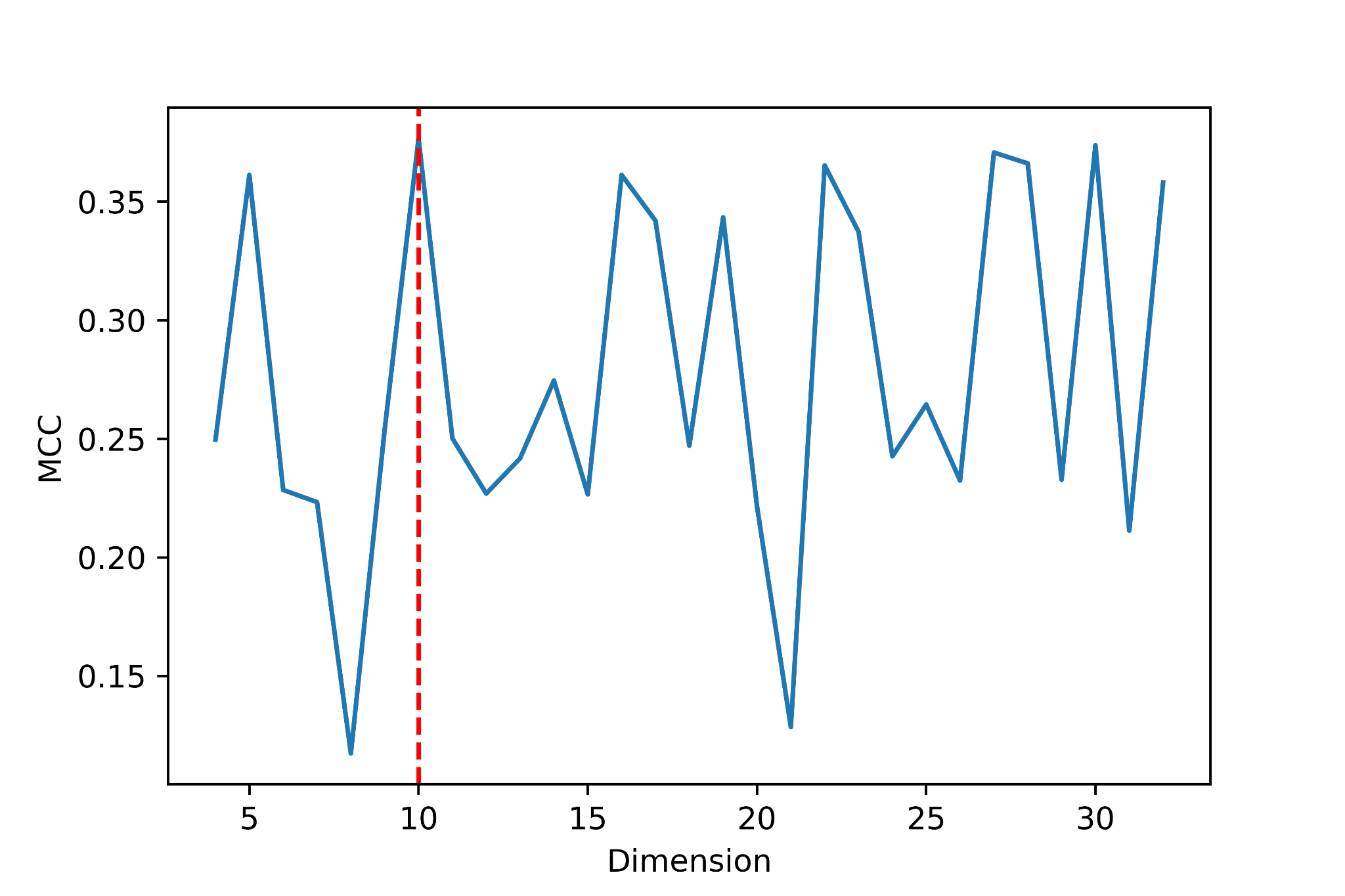  window size = 49, vocab size =23 |
| ConvLSTM | 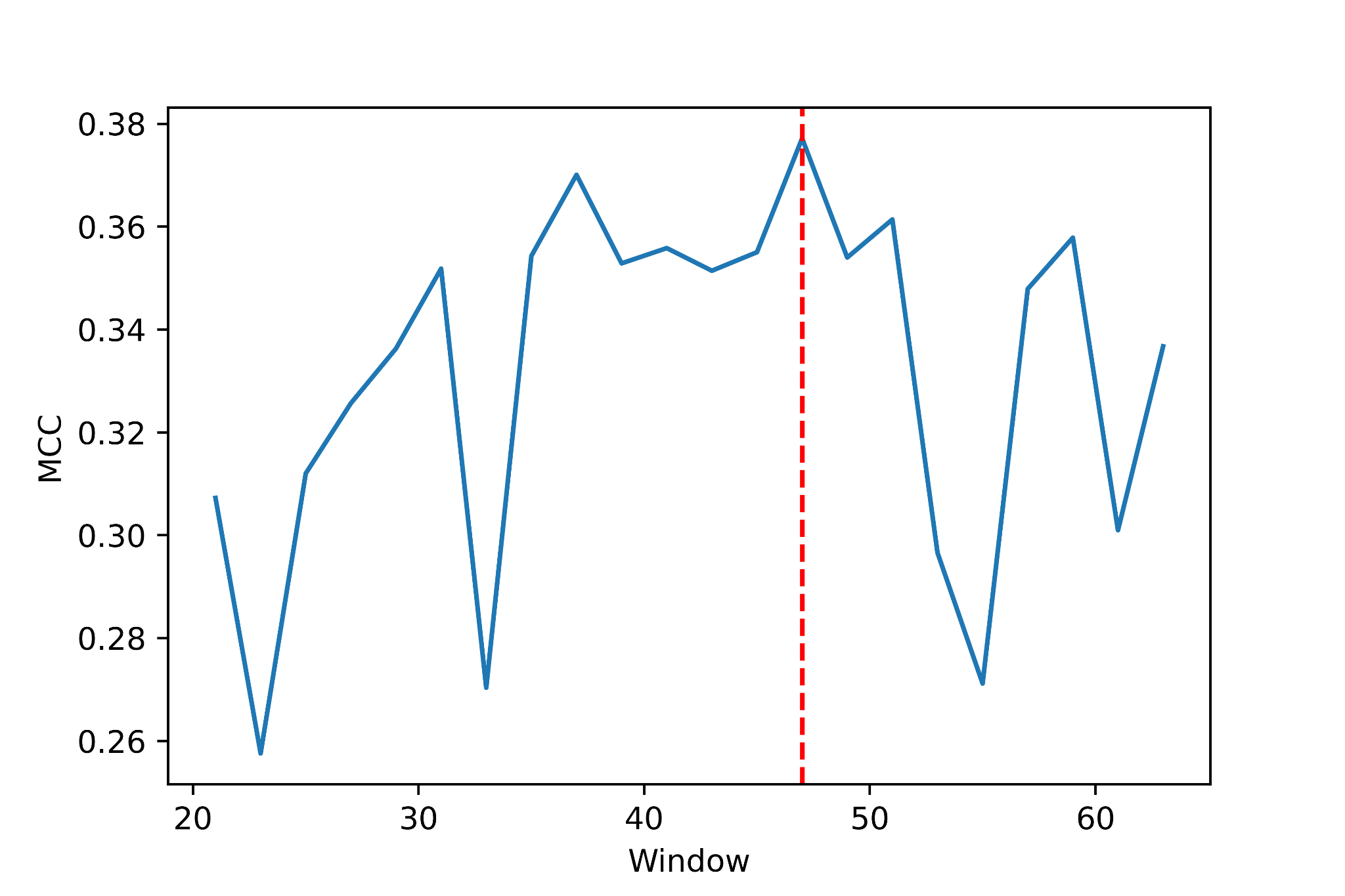  Embedding Dimension = 11, vocab size =23 | 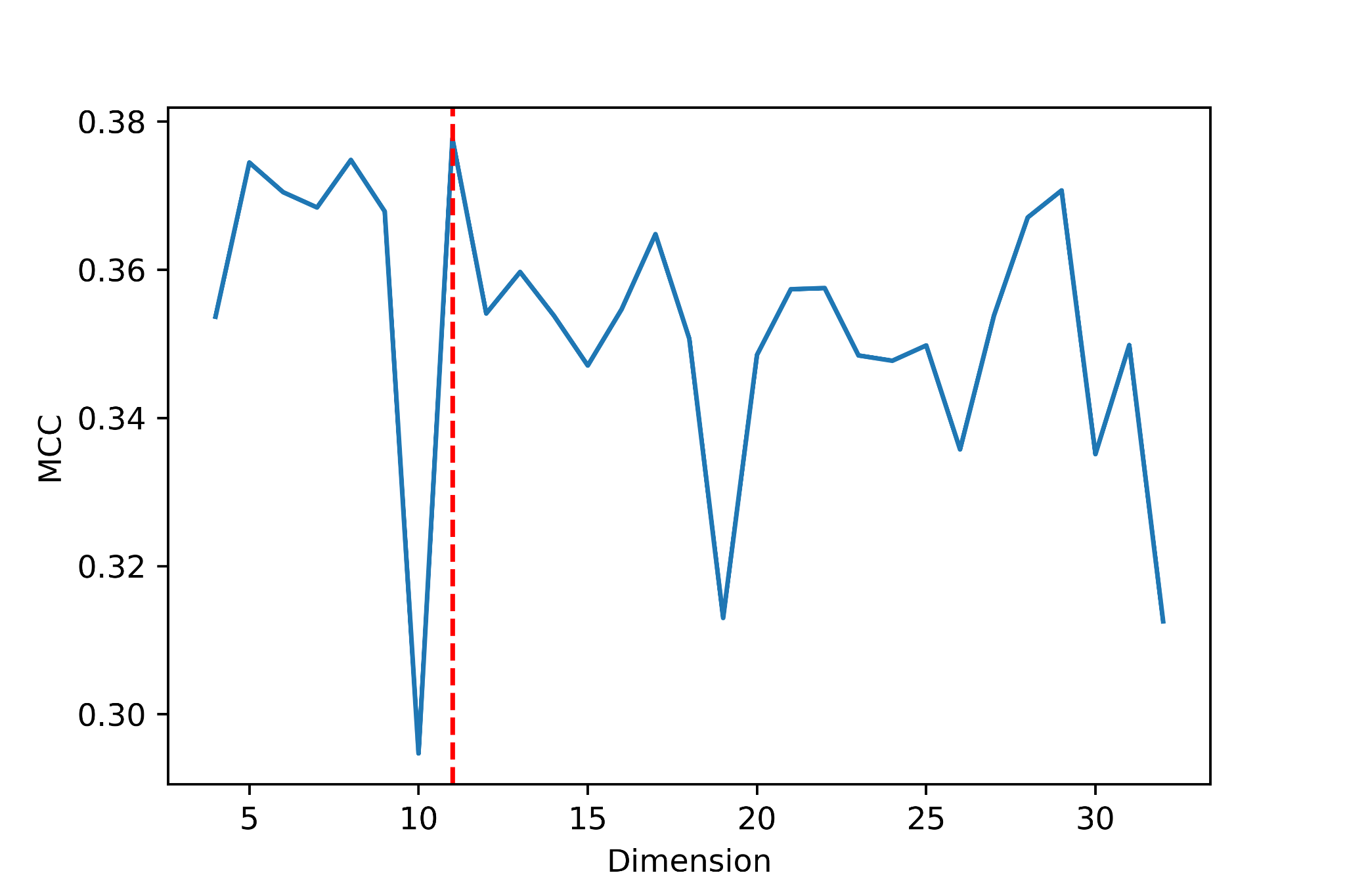  window size = 47, vocab size =23 |
| BiLSTM | 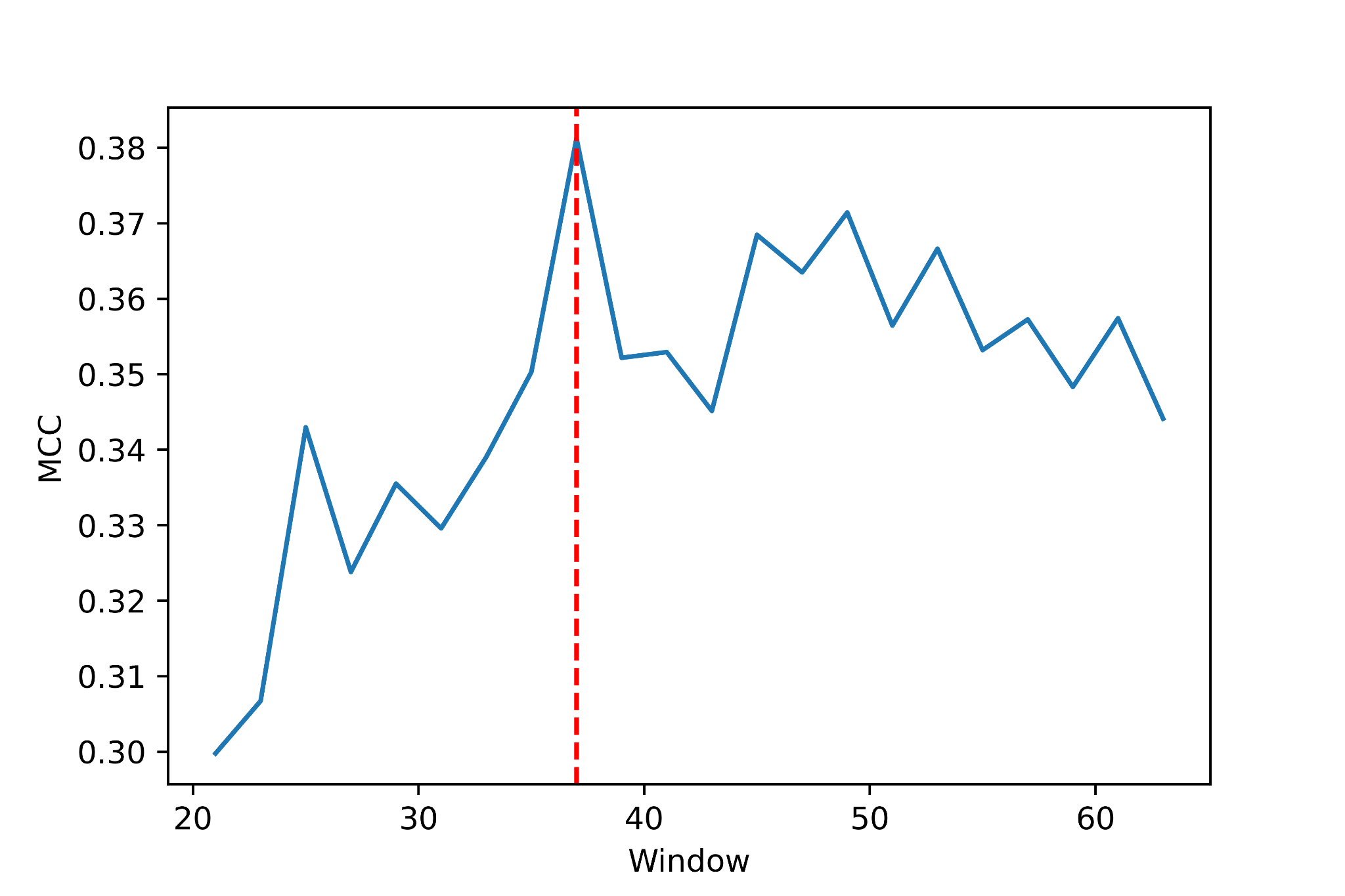  Embedding Dimension = 11, vocab size =23 | 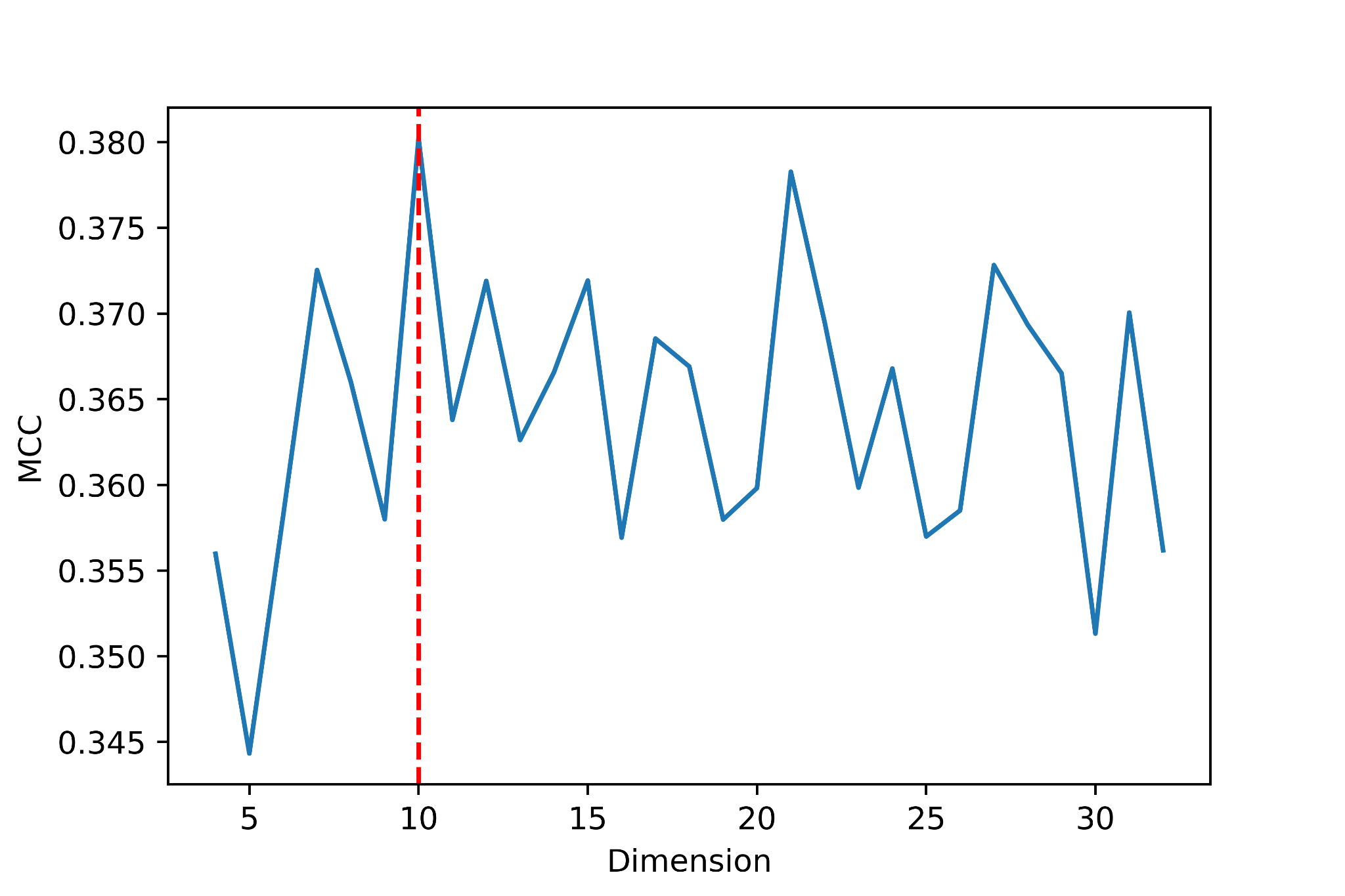  window size = 37, vocab size =23 |

**7. Comparison among different pLMs**

Besides, ProtT5 we also explored other two protein language models (ProtBert (ProtTrans family model, and ESM-1 model). 5-fold cross validation was used to find optimal architecture for each pLM representation. ANN was the best model among other architectures for all pLMs. Below we show the comparison of three pLMs based on independent set:

**Supplementary Table 6:** Independent test comparison of various pLMs

| **pLM** | **Model (optimal)** | **MCC** | **Sensitivity** | **Specificity** | **g-mean** | **AUC** |
| --- | --- | --- | --- | --- | --- | --- |
| ProtBERT | ANN | 0.20 | 0.48 | 0.80 | 0.62 | 0.64 |
| ESM-1 | ANN | 0.25 | 0.56 | 0.79 | 0.67 | 0.69 |
| ProtT5 | ANN | **0.29** | **0.60** | **0.81** | **0.70** | **0.71** |

It can be cocluded that ProtT5 performs better than the other compared pLMs.

**8. Frequency and WebLogo plots**

Additionally, we also plotted WebLogo for the train positive SNO sites and the train negative SNO sites. The results are shown in Supplementary Figure 2.

**Supplementary Figure 2:** Frequency and WebLogo plots for train positive and train negative window sequence (window size = 37)

| **Train Positive** | **Train Negative** |
| --- | --- |
| 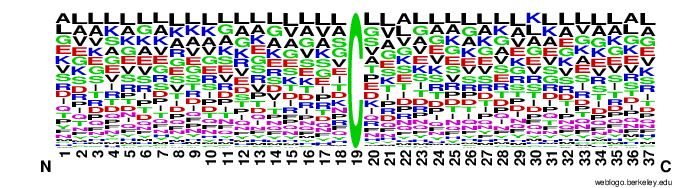 | 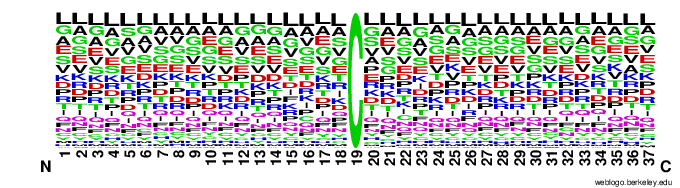 |
| 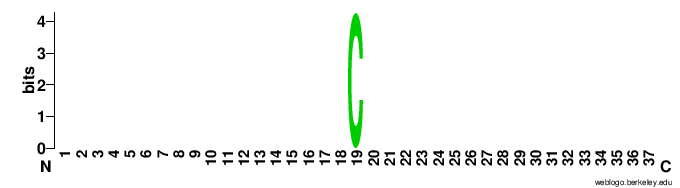 | 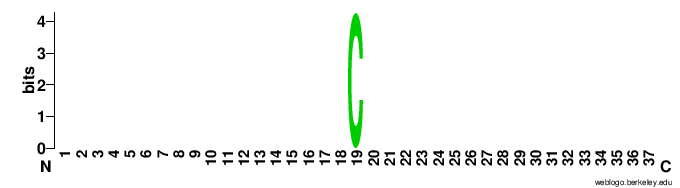 |

**9. PR Curves on independent test set**

**Supplementary Figure 3**: PR curves for base models and pLMSNOSite using a) imbalanced independent test set and b) using balanced independent test set

| 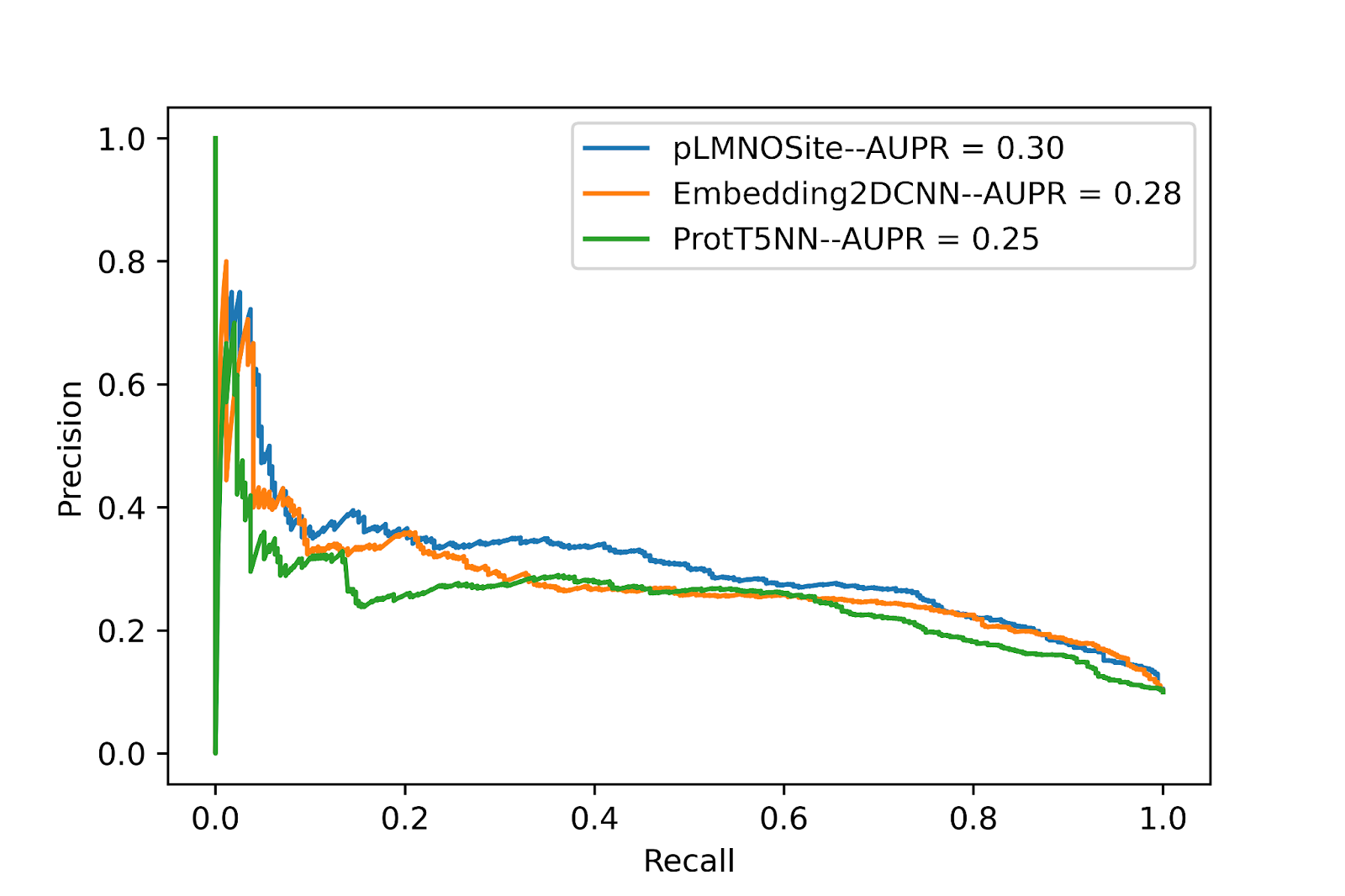  a) Using imbalanced independent test set | 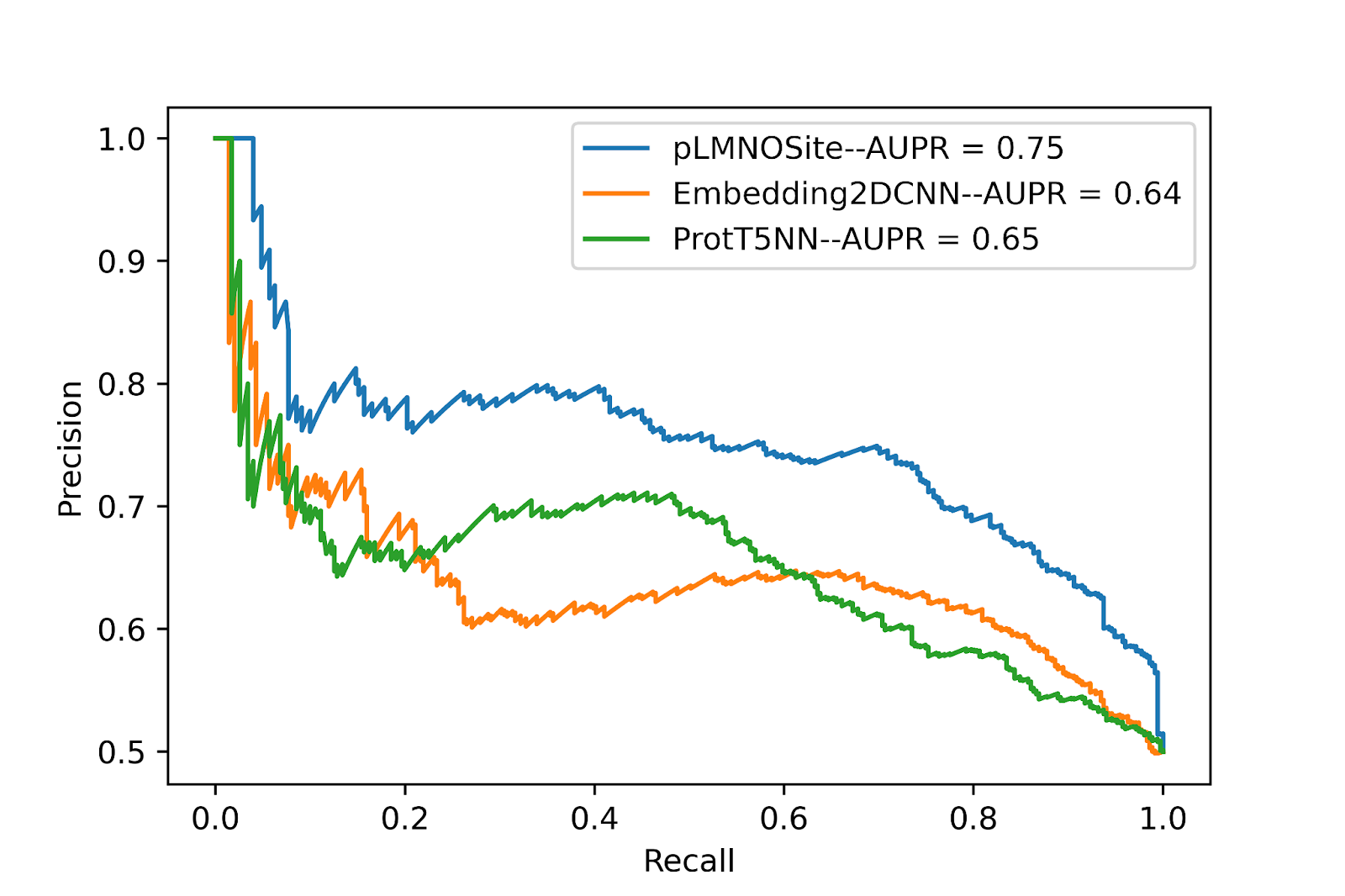  b) Using balanced independent test set |
| --- | --- |

**10. Precision Comparison of existing predictors with pLMNOSite**

**Supplementary Figure 4**: Comparison of pLMSNOSite with other existing predictors based on precision


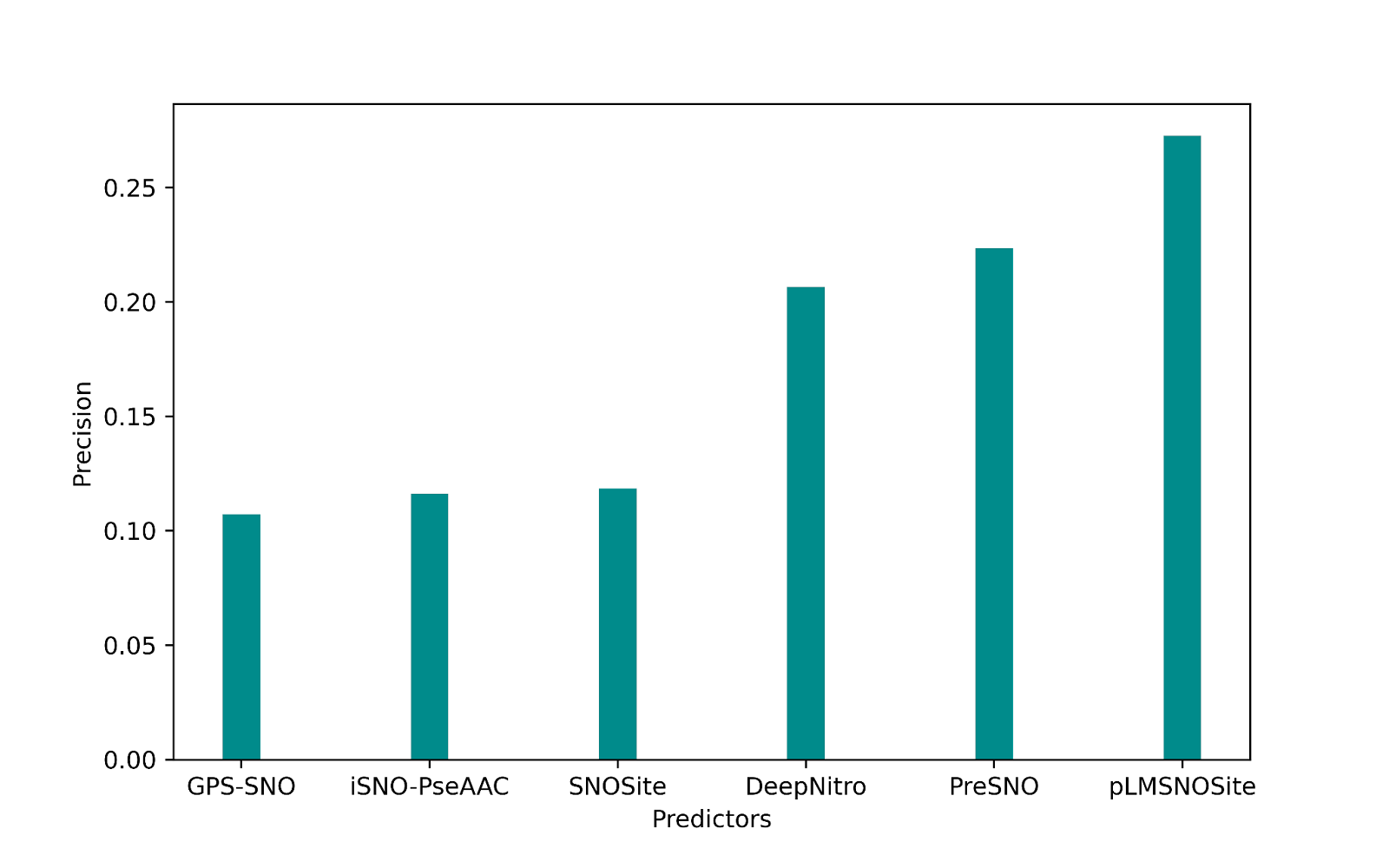

Supplement: Supplementary file 1 — Additional file 1. Contains supplementary tables and figures referred to in the manuscript. In sections 1, 2, and 3, we describe various ML/DL architectures and their respective hyperparameters. Table S1. Hyperparameter search space for models in the ProtT5 module. Table S2. Hyperparameter search space for models in the word embedding module. Table S3. Hyperparameter search space for models in the meta-classifier. Table S4. fivefold cross-validation results of Embedding2DCNN and ProtT5ANN when imbalanced learning (based on cost-sensitive learning) is performed. Table S5. Best combination (with respect to MCC) of window size and embedding dimension for each of the candidate models for the word embedding module on fivefold cross-validation. Figure S1. The sensitive analysis curves of each DL model in the word embedding module on fivefold cross-validation. Table S6. Comparison of ProtT5 with other pLMs such as ProtBERT (BERT-based ProtTrans family model) and Meta’s ESM-1 using independent testing. Figure S2. Frequency and WebLogo plots for train positive and train negative window sequences (window size = 37). Figure S3. Precision-Recall curves were produced for base models and pLMSNOSite using an imbalanced independent set and a balanced independent test set separately. Figure S4. Comparison of pLMSNOSite with other existing predictors based on precision values using an independent test set. [file 12859_2023_5164_MOESM1_ESM.docx]
